# Carbohydrate metabolism and fertility related genes high expression levels promote heterosis in autotetraploid rice harboring double neutral genes

**DOI:** 10.1186/s12284-019-0294-x

**Published:** 2019-05-10

**Authors:** Lin Chen, Yun Yuan, Jinwen Wu, Zhixiong Chen, Lan Wang, Muhammad Qasim Shahid, Xiangdong Liu

**Affiliations:** 1 State Key Laboratory for Conservation and Utilization of Subtropical Agro-Bioresources, South China Agricultural University, Guangzhou, 510642 China; 2 Guangdong Provincial Key Laboratory of Plant Molecular Breeding, South China Agricultural University, Guangzhou, 510642 China; 3 College of Agriculture, South China Agricultural University, Guangzhou, 510642 China

**Keywords:** Neo-tetraploid rice, Hybrid vigor, Transcriptome, Chromosome, Sterility, Polyploidy

## Abstract

**Background:**

Autotetraploid rice hybrids have great potential to increase the production, but hybrid sterility is a major hindrance in the utilization of hybrid vigor in polyploid rice, which is mainly caused by pollen abortion. Our previous study showed that double pollen fertility neutral genes, *Sa-n* and *Sb-n*, can overcome hybrid sterility in autotetraploid rice. Here, we used an autotetraploid rice line harboring double neutral genes to develop hybrids by crossing with auto- and neo-tetraploid rice, and evaluated heterosis and its underlying molecular mechanism.

**Results:**

All autotetraploid rice hybrids, which harbored double pollen fertility neutral genes, *Sa-n* and *Sb-n*, displayed high seed setting and significant positive heterosis for yield and yield-related traits. Cytological observations revealed normal chromosome behaviors and higher frequency of bivalents in the hybrid than parents during meiosis. Transcriptome analysis revealed significantly higher expressions of important saccharides metabolism and starch synthase related genes, such as *OsBEIIb* and *OsSSIIIa*, in the grains of hybrid than parents. Furthermore, many meiosis-related and specific genes, including *DPW* and *CYP703A3*, displayed up-regulation in the hybrid compared to a parent with low seed setting. Many non-additive genes were detected in the hybrid, and GO term of carbohydrate metabolic process was significantly enriched in all the transcriptome tissues except flag leaf (three days after flowering). Moreover, many differentially expressed genes (DEGs) were identified in the yield-related quantitative trait loci (QTLs) regions as possible candidate genes.

**Conclusion:**

Our results revealed that increase in the number of bivalents improved the seed setting of hybrid harboring double pollen fertility neutral genes. Many important genes, including meiosis-related and meiosis-specific genes and saccharides metabolism and starch synthase related genes, exhibited heterosis specific expression patterns in polyploid rice during different development stages. The functional analysis of important genes will provide valuable information for molecular mechanisms of heterosis in polyploid rice.

**Electronic supplementary material:**

The online version of this article (10.1186/s12284-019-0294-x) contains supplementary material, which is available to authorized users.

## Background

Heterosis, or hybrid vigor, is a complex biological phenomenon, which is improved or superior phenotypic performance of hybrid in comparison to one or both parents, such as enhanced grain yield, stress tolerance and biomass production. Heterosis has been extensively applied to increase the rice yield in the world (Cheng et al. 2007). However, productivity of rice has been stagnant in the past few years. Polyploid species play an important role in breeding programs, such as cotton (Flagel et al. 2008), wheat (Goncharov et al. 2007), and rapeseed (Albertin et al. 2006). Rice polyploidization is an effective method to increase the size of rice genome and improve the wide adaptability (Cai et al. 2001; Guo et al. 2017).

The polyploid rice hybrids showed stronger biological advantage and yield potential compared with diploid rice hybrids, and has attracted the attention of many rice researchers (Shahid et al. 2011; Wu et al. 2013; Guo et al. 2017). However, autotetraploid rice has many unfavorable traits, especially low seed setting, which limits its commercial utilization (Shahid et al. 2010, 2013a; Wu et al. 2014; Chen et al. 2018a). Recently, our research team found that polyploidy enhanced pollen sterility loci interactions and increased chromosomal abnormalities in autotetraploid hybrid rice (Wu et al. 2015), and also revealed that intersubspecific diploid and autotetraploid hybrid rice sterility could be overcome by double neutral genes (Shahid et al. 2013b; Wu et al. 2017). After years of efforts, our research group have developed few neo-tetraploid rice lines with high seed setting (> 80%) (Guo et al. 2016, 2017), and two new neo-tetraploid rice have been registered for the PVP (Protection for new varieties of plant) in China (Guo and Liu 2014). Moreover, neo-tetraploid rice could overcome the sterility and produce high heterosis in autotetraploid hybrid rice (Guo et al. 2017). Two photoperiod- and thermo-sensitive genic male sterile lines (PS006 and PS012) of polyploid rice showed stronger hybrid vigor and great potential for improving rice quality and productivity (Zhang et al. 2017).

Next generation high-throughput sequencing, such as RNA sequencing (RNA-seq) and microarray technology, is widely used to investigate gene expression and function. The RNA-seq enabled us to understand differentially expressed genes associated with abiotic stresses and pollen development in diploid rice (Jin et al. 2013; Hu et al. 2016; Fu et al. 2017). The RNA-seq has also been applied to detect differentially expressed genes between diploid and autotetraploid rice during pollen development (Wu et al. 2014; Chen et al. 2018a; Li et al. 2018). Furthermore, RNA-seq has been widely used to investigate heterosis in various plants, such as wheat (Liu et al. 2018), maize (Ma et al. 2018), rapeseed (Shen et al. 2017), tobacco (Tian et al. 2018) and rice (Wei et al. 2009). The differentially expressed genes were found to be closely associated with heterosis in super rice LYP9 and its parents through RNA-seq (Wei et al. 2009). Later, many differentially expressed genes closely related to root heterosis at tillering and heading stage were detected in super hybrid XY9308 and its parents through RNA-seq (Zhai et al. 2013). Chen et al. (2018b) compared the transcriptomes between super hybrid Wufengyou T025 (WFYT025) and its parents during young panicle development, and suggested that carotenoid biosynthesis and plant hormone signal transduction were enriched in differentially expressed genes, and these genes were related to the grain number heterosis. A number of genes associated with leaf, anthers and ovary heterosis in neo-tetraploid rice hybrids were identified by RNA-seq, which were related to photosynthesis and metabolic process and transport (Guo et al. 2017).

Our previous study indicated that saccharide abnormal distribution and down-regulation of saccharide transport genes may cause pollen sterility and lead to low seed setting in autotetraploid rice (Chen et al. 2018a). In this study, to increase yield of autotetraploid rice, we used an autotetraploid rice line harboring double neutral genes for pollen fertility at *Sa* and *Sb* loci, which could overcome F_1_ sterility when it crossed with low fertility autotetraploid rice (Wu et al. 2017; Chen et al. 2018a). We thus primarily aimed to evaluate heterosis mechanism of neo-tetraploid and autotetraploid rice harboring double neutral genes, and to observe the role of chromosome configuration and behavior in heterosis and fertility. In addition, we detected differentially expressed genes between parents and hybrid in nine tissues at three development stages using RNA-seq, which would provide insights into the molecular mechanism underlying heterosis in autotetraploid and neo-tetraploid rice, and provide new germplasm for polyploid rice breeding.

## Results

### Heterosis evaluation of hybrids generated by crossing of autotetraploid with neo-tetraploid rice

Analysis of the agronomic traits of five hybrids, which were developed by crossing autotetraploid rice line (T449) with five neo-tetraploid rice lines, showed significant improvement in important yield-related traits, including number of filled grains per plant, yield per plant and seed setting. Evaluation of heterosis indicated that the values for mid-parent heterosis (MPH) were positive for all the traits except grain length and total grains per plant, and the highest MPH was found for grain yield per plant (170.89%). The high-parent heterosis (HPH) values were positive for the filled grains and grain yield per plant, and the highest HPH was detected for filled grains per plant (71.10%) (Additional file 1: Table S1).

**Fig. 1:**
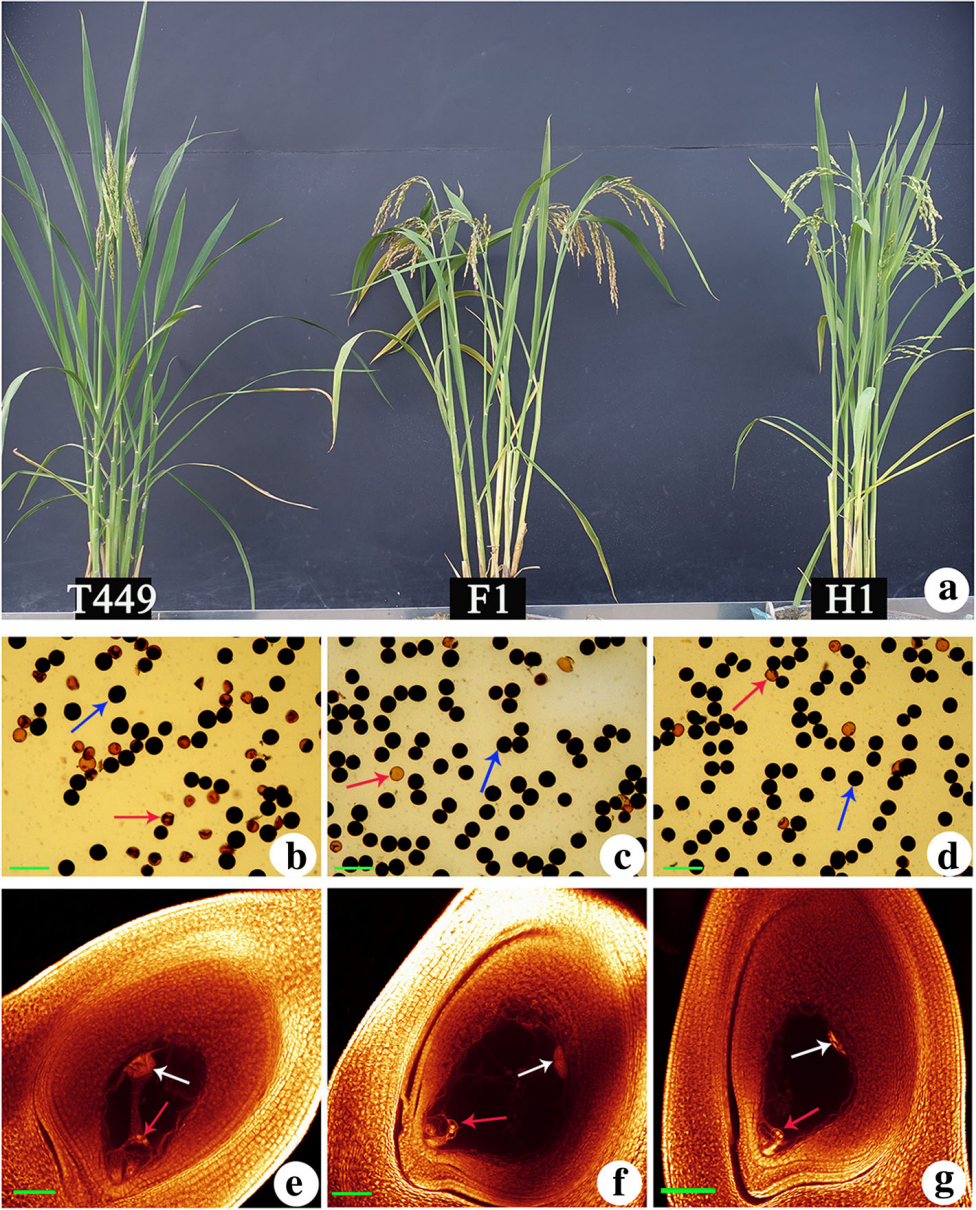
Comparisons of morphological characteristics between F_1_ hybrid and its parents. **a** Plant appearance of F_1_, T449 and H1. Pollens of T449 (**b**), F_1_ (**c**), and H1 (**d**), blue arrows indicate normal pollens, red arrows indicate abnormal pollens. Bar = 100 μm. Embryo sacs of T449 (**e**), F_1_ (**f**) and H1 (**g**), white arrows indicate antipodal cells and red arrows indicate two polar nuclei. Bar = 100 μm

Then, we further selected a hybrid (T449 × H1) for transcriptome analysis to analyze the heterosis mechanism in detail. The hybrid had high seed setting, pollen and embryo sac fertility (Fig. 1; Table 1 and Additional file 2: Table S2), although the maternal line, T449, had low pollen fertility and seed setting. The hybrid displayed significant positive MPH for all the traits, and the values for MPH were very high for filled grains per plant, grain yield per plant and seed setting (Table 1). Meanwhile, the hybrid also showed positive HPH values for most of the traits except effective number of panicles per plant, 1000-grain weight, and grain length and width (Table 1).

**Table 1:** Heterosis analysis of hybrids generated by crossing of T449 and neo-tetraploid (H1) rice lines

Traits	T449	H1	F_1_	MPH (%)	HPH (%)
PH (cm)	87.40 ± 3.91	102.50 ± 3.09	116.44 ± 3.74	22.63	13.60
EP	4.40 ± 0.55	7.60 ± 1.34	6.60 ± 1.34	10.00	−13.16
GL (cm)	8.07 ± 0.11	8.92 ± 0.12	8.80 ± 0.16	3.59	−1.35
GW (cm)	2.71 ± 0.15	3.23 ± 0.05	3.10 ± 0.09	4.38	−4.02
TGW (g)	30.20 ± 0.89	36.85 ± 0.56	36.15 ± 0.41	7.83	−1.90
FGP	127.00 ± 31.89	421.20 ± 49.26	757.00 ± 166	176.18	79.72
TGP	612.00 ± 100.21	614.60 ± 134.33	907.80 ± 179.58	48.02	47.71
GYP (g)	3.83 ± 0.93	15.53 ± 1.90	27.32 ± 5.79	182.36	75.98
SS (%)	20.55 ± 2.63	69.75 ± 7.79	83.12 ± 2.83	84.10	19.17
PF (%)	35.10 ± 6.28	77.95 ± 2.26	77.01 ± 0.47	36.24	−1.21

Note: Fifteen plants were selected for traits investigations; HPH: High-parent heterosis; MPH: Mid-parent heterosis. PH: Plant height; EP: Effective number of panicles per plant; GL: Grain length; GW: Grain width; TGW: 1000-grain weight; FGP: Filled grains per plant; TGP: Total grains per plant; GYP: Grain yield per plant; SS: Seed setting; PF: Pollen fertility; ±: represents standard deviation

### Chromosome configuration at diakinesis and metaphase I in F_1_ hybrid compared to its parents

**Fig. 2:**
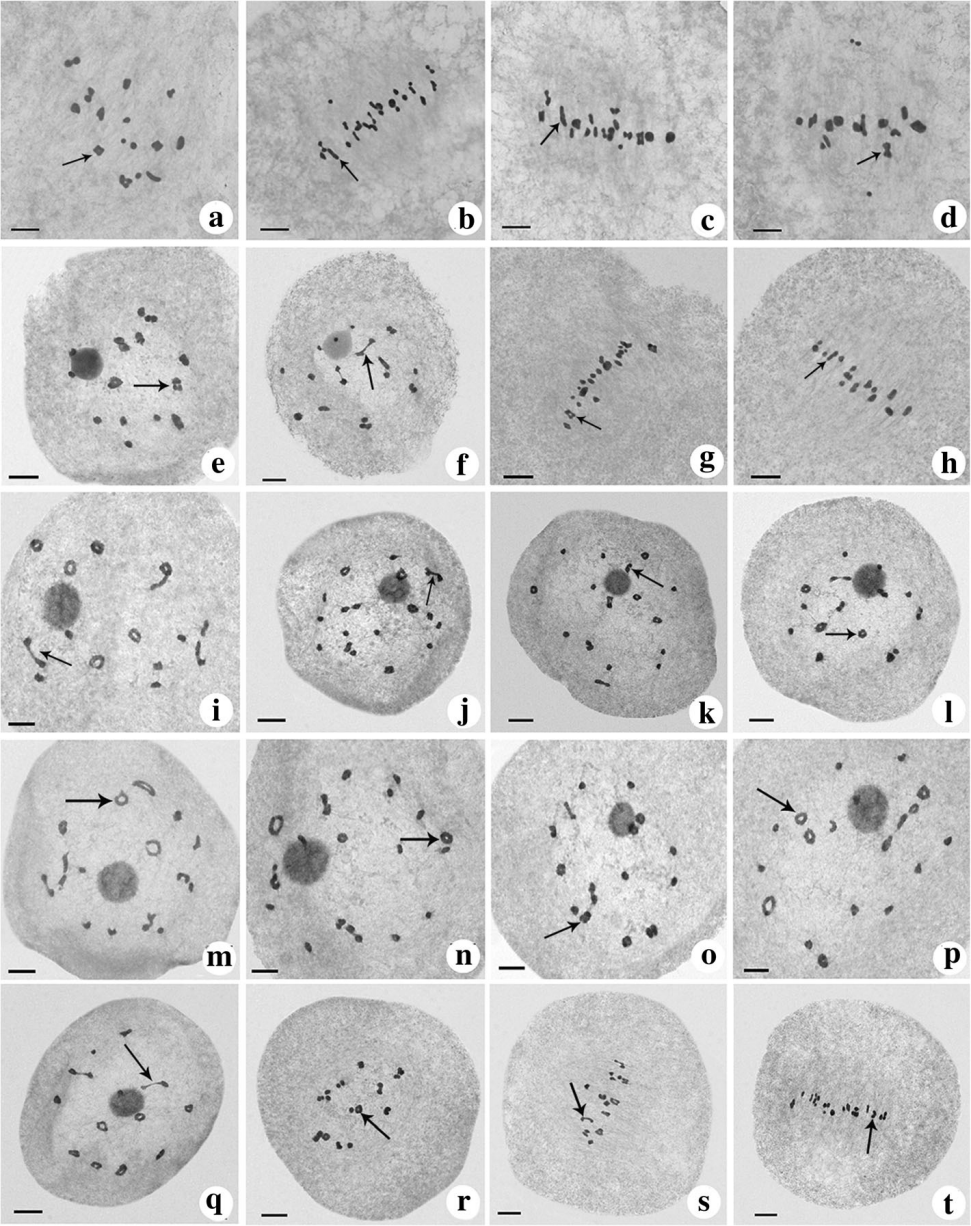
Chromosome configuration at diakinesis and metaphase I. **a**-**d**, T449. E-H, H1. I-T, F_1_. **a**, 9IV (6 ring shape (arrow) + 1 OK shape + 2 X shape) + 2III + 2II + 2I; **b**, 2IV (1 ring shape + 1 chain shape (arrow)) + 2III + 14II + 6I; **c**, 6IV (4 ring shape + 1 chain shape (arrow) + 1 X shape) + 3III + 7II + 1I; **d**, 9IV (7 ring shape + 1 chain shape + 1 X shape (arrow)) + 3III + 1II + 1I; **e**, 9IV (6 ring shape + 1 OK shape (arrow) + 2 X shape) +6II; **f**, 8IV (1 ring shape + 1 chain shape+ 2 frying pan shape (arrow) + 4 X shape) +8II; **g**, 7IV (4 ring shape (arrow) + 1 OK shape + 2X shape) +8II + 4I; **h**, 9IV (5 ring shape + 4 X shape + 1 chain shape (arrow)) +6II; **i**, 9IV (6 ring shape + 2 frying pan shape (arrow) + 1 chain shape) + 6II; **j**, 6IV (4 ring shape + 1 frying pan shape (arrow) + 1 X shape) + 12II; **k**, 8IV (4 ring shape + 1 chain shape + 3 X shape (arrow)) + 8II; **l**, 8IV (5 ring shape (arrow) + 3 X shape) + 8II; **m**, IV (3 ring shape (arrow) + 1 frying pan shape + 1 OK shape + 1 chain shape + 2 X shape) + 8II; **n**, 4IV (4 ring shape (arrow)) + 15II + 2I;**o**, 8IV (5 ring shape + 1 frying pan shape + 1 OK shape (arrow) + 1 X shape) +8II; **p**, 5IV (5 ring shape (arrow)) + 14II; **q**, 11IV (6 ring shape + 2 frying pan shape (arrow) + 1 OK shape+ 2 X shape) + 2II; **r**, 8IV (3 ring shape (arrow) + 5 X shape) + 8II; **s**, 8IV (4 ring shape + 1 frying pan shape (arrow) + 3 X shape) + 8II; **t**, 6IV (4 ring shape +2 X shape (arrow)) + 11II+ 2I. Bar = 10 μm

**Fig. 3:**
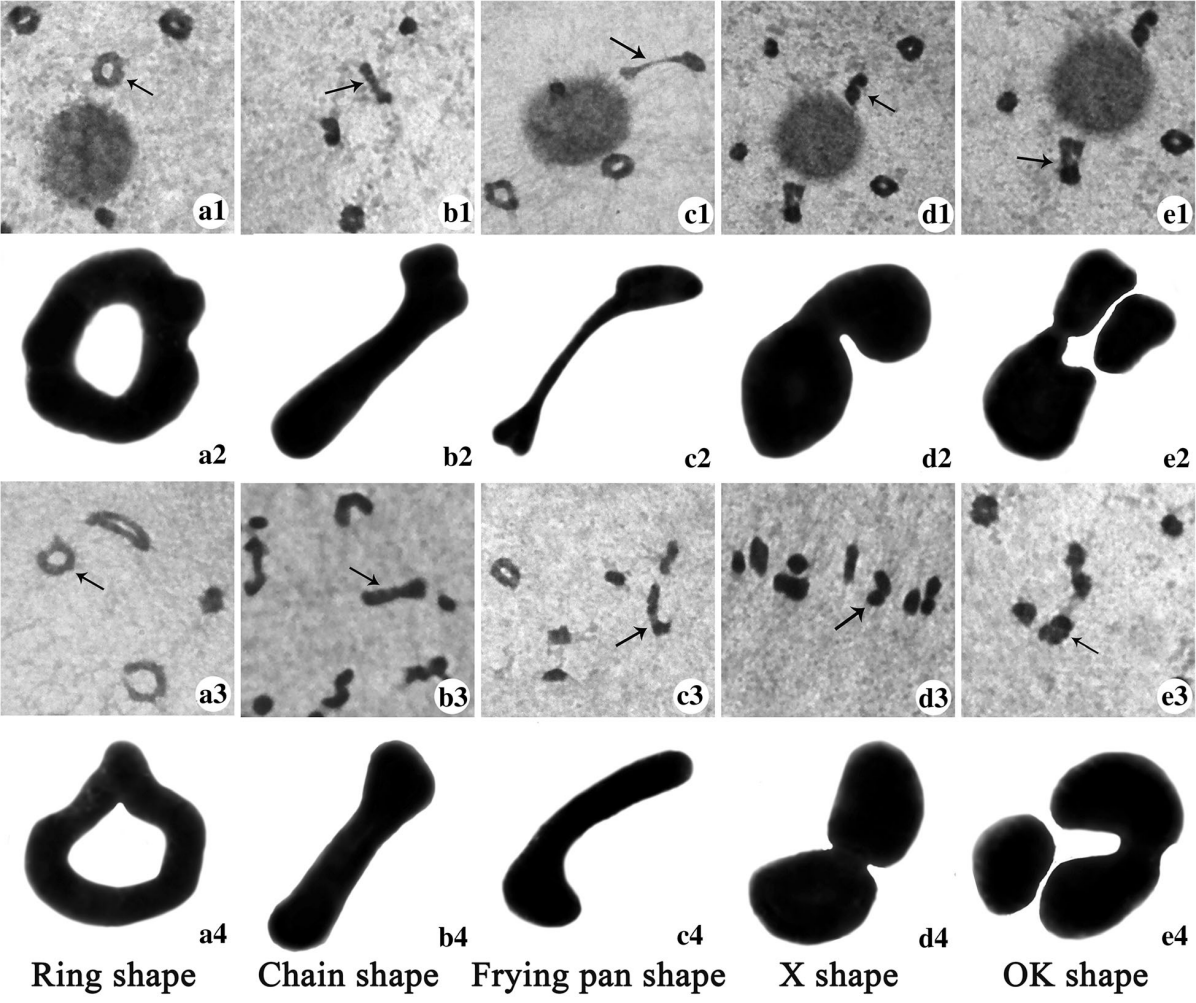
Classification of chromosome configuration. a1–a4 represent ring shape, b1–b4 represent chain shape, c1–c4 represent frying pan shape, d1–d4 represent “X” shape, e1–e4 represent “OK” shape

The tetravalent was the most type of chromosome configuration at diakinesis and metaphase I in the parents. The numbers of bivalent chromosomes were higher in H1 (high seed setting) than T449 (low seed setting), and univalent type of chromosomes were higher in T449 than both H1 and F_1_ (Table 2, Fig. 2). However, bivalent chromosomes were the most frequent in F_1_ (Table 2), and 9.07 and 12.57 bivalents were found in each pollen mother cell (PMC) at diakinesis and metaphase I, respectively. There were significant differences in the numbers of chromosome configurations between F_1_ hybrid and parents, and these results indicated that the increase of bivalent type may improve pollen fertility and seed setting. The chromosome configurations of tetravalent types were divided into five types, including ring shape (Fig. 3a1-a4), chain shape (Fig. 3b1-b4), frying pan shape (Fig. 3c1-c4), “X” shape (Fig. 3d1-d4) and “OK” shape (Fig. 3e1-e4). The frequency of ring shape was the most frequent chromosome configuration of tetravalent types at diakinesis and metaphase I (Table 2).

**Table 2:** Meiotic chromosome configurations at diakinesis and metaphase I in F_1_ hybrid and parents

Material	Stage	No.	I	II	III	IV					
						ring shape	chain shape	X shape	frying pan shape	OK shape	Total
T449	Diakinesis	124	3.21 ± 2.87	5.73 ± 4.30	1.23 ± 1.50	2.91 ± 1.84	1.03 ± 1.04	2.13 ± 1.60	0.81 ± 1.03	0.52 ± 0.78	7.41 ± 2.02
	Metaphase I	53	4.13 ± 3.15	5.36 ± 3.42	1.26 ± 1.30	3.06 ± 1.94	1.28 ± 1.23	2.08 ± 1.48	0.49 ± 0.72	0.43 ± 0.69	7.34 ± 2.23
H1	Diakinesis	135	0.59 ± 1.41	6.10 ± 3.91	1.28 ± 2.29	4.79 ± 2.20	0.44 ± 0.80	2.33 ± 1.93	0.15 ± 0.41	0.14 ± 0.37	7.84 ± 2.13
	Metaphase I	53	2.51 ± 7.50	6.53 ± 4.52	0.92 ± 2.08	4.06 ± 2.28	0.43 ± 0.82	2.53 ± 1.85	0.17 ± 0.47	0.23 ± 0.42	7.42 ± 2.80
F_1_	Diakinesis	98	0.51 ± 1.52	9.07 ± 4.45	0.08 ± 0.31	4.42 ± 2.15	0.28 ± 0.53	2.30 ± 1.53	0.18 ± 0.54	0.09 ± 0.29	7.28 ± 2.28
	Metaphase I	99	0.65 ± 1.19	12.57 ± 5.36	0.08 ± 0.27	1.95 ± 1.79	0.42 ± 0.72	2.76 ± 1.61	0.14 ± 0.53	0.21 ± 0.44	5.49 ± 2.65

Note: No. represents the number of cells. I, II, III and IV represent univalent, bivalent, trivalent and tetravalent, respectively. Each number in columns I - IV represents the average number of chromosome configuration ± SD / cell

### Chromosome behavior during PMC meiosis in F_1_ hybrid and its parents

Meiotic stages in hybrid were consistent with its parents, and could be divided into nine development stages, including prophase I (leptotene, zygotene, pachytene, diplotene, and diakinesis) (Additional file 3: Figure S1A-D), metaphase I (Additional file 3: Figure S1E), anaphase I (Additional file 3: Figure S1F), telophase I (Additional file 3: Figure S1G), prophase II (Additional file 3: Figure S1H), metaphase II (Additional file 3: Figure S1I), anaphase II (Additional file 3: Figure S1J), telophase II (Additional file 3: Figure S1K), and tetrad (Additional file 3: Figure S1L). The average frequency of normal chromosome behavior at metaphase I, anaphase I, telophase I, metaphase II, anaphase II, telophase II were remarkably higher in F_1_ and H1 than T449 (Table 3 and Additional file 5: Table S3).

**Table 3:** Frequency of normal chromosome behaviors during meiosis in hybrid and parents

	T449		H1		F_1_	
Stage	Number of cells	Normal (%)	Number of cells	Normal (%)	Number of cells	Normal (%)
Metaphase I	243	72.02	245	98.37	173	83.82
Anaphase I	106	61.32	141	94.33	83	87.95
Telophase I	170	77.06	215	99.53	144	96.53
Metaphase II	294	58.84	145	90.34	183	65.57
Anaphase II	139	23.02	50	44.00	75	32.00
Telophase II	249	67.07	179	94.41	129	92.25
Tetrad stage	1266	98.03	850	98.94	888	98.99

### Differentially expressed genes in F_1_ hybrid and parents

In order to investigate transcriptome changes in F_1_ and its parents, the transcriptome profiles of F_1_ and parents were analyzed in nine tissues, including anthers (P1) and flag leaves (L1) at meiosis stage, and flag leaves (L2), leaf sheath (Z2), anther (P2) and embryo sac (E2) at pre-flowering stage, and flag leaves (L3), leaf sheath (Z3) and grain (P3) at three days after flowering (Additional file 4: Figure S2). In total, more than 3.7 billion clean reads were detected in different samples. We aligned clean reads against the Nipponbare reference genome (MSU 7.0), and 92.52% to 96.32% annotated transcripts of the reference genome was obtained in our materials (Additional file 6: Table S4). The correlation for the gene expression level from three biological replicates of each line was more than 0.8 (Additional file 7: Table S5), and principal component analysis (PCA) indicated that three biological replicates were clustered together, and flag leaves and leaf sheath were also clustered together (Additional file 8: Figure S3). The correlations between F_1_ and its parents were investigated in different samples by hierarchical cluster analysis using Cluster 3.0 software. The results demonstrated that F_1_ and its parents always assembled into primary groups at the same tissue, and the transcriptome profiles of F_1_ were similar to H1, and these results were consist with the morphological and cytological observations (Additional file 9: Figure S4). A total of 12 DEGs were randomly selected for qRT-PCR validation. We compared the qRT-PCR results, and the expression trends were consistent with RNA-seq data, and a correlation coefficient was R^2^ = 0.8806 (Additional file 10: Figure S5), which demonstrated that RNA-seq data is reliable.

### Identification of differentially expressed genes (DEGs) by RNA-Seq

**Fig. 4:**
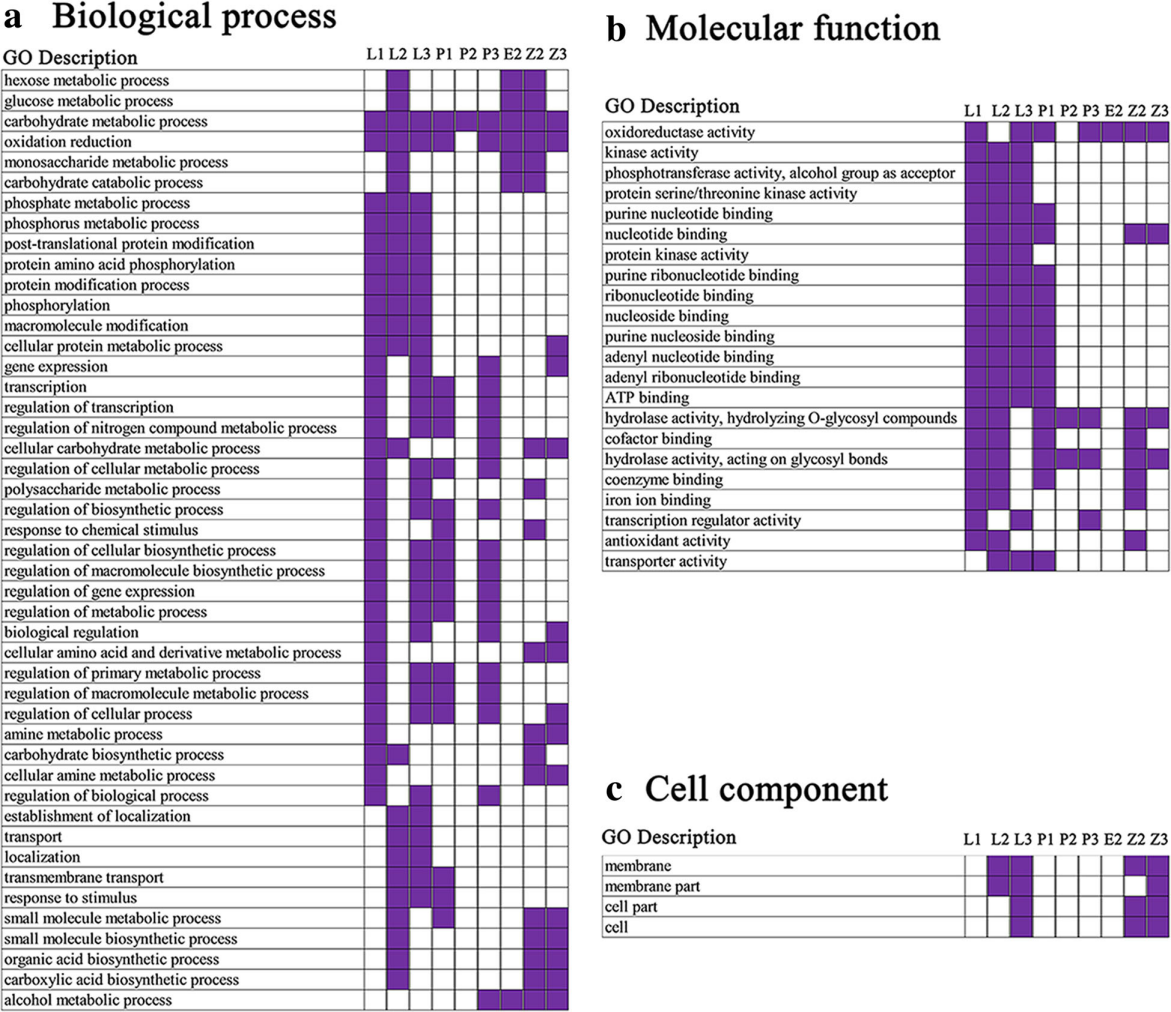
Gene ontology (GO) enrichment heat map for DEG_FPU_ in 9 tissues (GO terms were selected based on their appearance at least in three tissues or more). L1 and P1 represent flag leaves and anthers at meiosis stage, respectively. L2, P2, E2 and Z2 represent flag leaves, anther, embryo sac and leaf sheath at pre-flowering stage, respectively. L3, P3 and Z3 represent flag leaves, grain and leaf sheath at three days after flowering, respectively

The two filter conditions (false discovery rate (FDR) less than or equal to 0.05 and fold change (FC) higher than or equal to 2) were applied to identify DEGs. Using these two filter conditions, we identified 781 to 3813 DEGs in different tissues between F_1_ compared to its parents and between two parents (Table 4). We defined DEGs between the hybrid and its parents as DEG_FP_ and those between the parental lines as DEG_PP_. The DEG_FP_ can be divided into two categories, such as DEG_C_ that were shared by DEG_PP_ and DEG_FP_, and another uniquely belonging to F_1_ compared to parents, which were called as DEG_FPU_. The DEG_FPU_ may relevant to phenotypic differences between F_1_ and its parents (Wei et al. 2009); therefore, we specifically focused on the DEG_FPU_ to explore genes associated with the heterosis in polyploid rice. A total of 984 DEGs belonging to 50 transcription factor (TF) families, including 188, 102, 95, 148, 92, 96, 56, 84 and 123 TFs, were found in the L1, L2, L3, P1, P2, P3, E2, Z2 and Z3, respectively. The TFs of bHLH, WRKY, WRKY, NAC, NAC, NAC, ERF, MADS and bHLH were mostly detected in L1, L2, L3, P1, P2, P3, E2, Z2 and Z3, respectively (Additional file 11: Figure S6). The gene ontology (GO) enrichment analysis was employed for the functional categorization of genes, and the results revealed that differential gene expressions might be associated with fertility and heterosis in flag leaf, leaf sheath, anther, embryo sac and grain. GO analysis showed that a total of 68, 69, 79, 36, 4, 40, 16, 103 and 64 GO terms were significantly enriched in L1, L2, L3, P1, P2, P3, E2, Z2 and Z3, respectively. Interestingly, GO term of carbohydrate metabolic process was significantly enriched in all tissues (Fig. 4 and Additional file 12: Table S6). To further understand the functions of DEG_FPU_, we classified these genes according to their functional categories by KEGG pathway analysis, and the results showed that DEG_FPU_ were mostly enriched in the carbohydrate metabolism categories (Table 5). A total of 79, 60, 37, 70, 27, 58, 33, 78 and 93 DEG_FPU_ were involved in the carbohydrate metabolism categories in L1, L2, L3, P1, P2, P3, E2, Z2 and Z3, respectively.

**Table 4:** Number and classification of differentially expressed genes (DEGs) in parents and F_1_ hybrid harboring double neutral genes

Sample	DEG_PP_	T/F_1_	H/F_1_	DEG_FPU_	DEG_C_	DEG_FP_
L1	3813	3485	3041	2693	2549	5242
L2	3576	3264	1342	1740	2428	4168
L3	1886	1419	1402	1200	1261	2461
P1	1413	2054	1681	2314	457	2771
P2	2280	2556	1075	1504	1654	3158
P3	1866	1520	1561	1424	1109	2533
E2	1439	1479	781	927	1037	1964
Z2	1664	2051	1168	1631	1110	2741
Z3	2075	3051	1440	2505	1504	4009

Note: T, H, and F_1_ refer to T449, H1, and hybrid (F_1_), respectively. DEG_PP_ refers to DEG between T449 and H1, DEG_FP_ refers to DEG between hybrid and its parents. DEG_FPU_ denotes the unique numbers of DEG_FP_ that were belong to F_1_ only, and DEG_C_ denotes the overlapped genes between DEG_PP_ and DEG_FP_. L1 and P1 represent flag leaves and anthers at meiosis stage, respectively. L2, P2, E2 and Z2 represent flag leaves, anther, embryo sac and leaf sheath at pre-flowering stage, respectively. L3, P3 and Z3 represent flag leaves, grain and leaf sheath at three days after flowering, respectively

**Table 5:** Functional classification of DEG_FPU_ in different tissues

Functional categories	L1	L2	L3	P1	P2	P3	E2	Z2	Z3
Metabolism									
Amino acid metabolism	61*	38*	28*	49**	18	33*	11	33	51
Biosynthesis of other secondary metabolites	55**	29*	18	31*	14	17	15**	30*	42
Carbohydrate metabolism	79	60*	37	70**	27	58**	33**	78*	93*
Energy metabolism	17	37*	18	20	18	21	12	47*	61**
Glycan biosynthesis and metabolism	4	2	2	8	4	2	1	4	9
Lipid metabolism	42	26	21	41**	18	24	4	30	37
Metabolism of cofactors and vitamins	20	31**	14	25*	7	12	6	22	22
Metabolism of other amino acids	31*	16	11	23*	9	11	7	30**	16
Metabolism of terpenoids and polyketides	26	21**	11	15	9	12	7	25**	29*
Nucleotide metabolism	23	10	5	7	5	7	4	6	20
Genetic Information Processing									
Replication and repair	13	6	3	6	3	2	0	1	10
Transcription	17	6	8	11	8	13	2	6	18
Translation	79	10	10	27	25	27	7	37	124**
Folding, sorting and degradation	35	23	16	34	18	26	12	33	43
Environmental Information Processing									
Signal transduction	52**	27	22*	19	10	19	15*	17	31
Membrane transport	2	1	1	2	0	2	0	3	2
Cellular Processes									
Transport and catabolism	29	19	11	27	11	8	4	16	32
Organismal Systems									
Environmental adaptation	50**	40**	26**	30	22**	10	11	23	32
Human Diseases									
Endocrine and metabolic diseases	3	2	3	2	1	1	1	0	1

* and ** denote significant enrichment of DEG_FPU_ among functional categories with *P* < 0.05 and *P* < 0.01, respectively. L1 and P1 represent flag leaves and anthers at meiosis stage, respectively. L2, P2, E2 and Z2 represent flag leaves, anther, embryo sac and leaf sheath at pre-flowering stage, respectively. L3, P3 and Z3 represent flag leaves, grain and leaf sheath at three days after flowering, respectively

### Gene expression patterns of DEG_FP_ and non-additive genes (NAGs) in the hybrid

According to the gene expression levels in F_1_ hybrid relative to its parents, the DEG_FP_ were classified into five groups, including higher than both parents (HBP), close to higher parent (CHP), between both parents (BBP), close to lower parent (CLP) and lower than both parents (LBP). Genes classified as HBP accounted for the majority of DEG_FP_ in P1, CHP accounted for the majority of DEG_FP_ in L3 and P3, CLP occupied the majority of DEG_FP_ in the L1, L2, P2, E2, Z2 and Z3, while the BBP and LBP groups accounted for the smallest number of genes in all tissues (Table 6). These results showed that most of the DEG_FP_ were HBP, CHP and CLP in the hybrid, which revealed that these genes would have an important role in the heterosis.

**Table 6:** Number and classification of DEG_FP_

	HBP	a%	CHP	b%	BBP	c%	CLP	d%	LBP	e%
L1	1433	27.34	1418	27.05	215	4.10	1854	35.37	322	6.14
L2	357	8.57	1011	24.26	228	5.47	2228	53.45	344	8.25
L3	225	9.14	925	37.59	102	4.14	784	31.86	422	17.15
P1	1071	38.65	693	25.01	148	5.34	591	21.33	269	9.71
P2	363	11.49	843	26.69	237	7.50	1397	44.24	318	10.07
P3	543	21.44	785	30.99	135	5.33	741	29.25	329	12.99
E2	246	12.53	650	33.10	71	3.62	754	38.39	243	12.37
Z2	397	14.48	807	29.44	151	5.51	967	35.28	419	15.29
Z3	676	16.86	1156	28.84	132	3.29	1692	42.21	353	8.81

DEG_FP_ was classified into five groups according to their expression levels relative to both parental inbred lines. HBP, higher than both parents, CHP, close to high parent, BBP, between two parents, CLP, close to lower parent, LBP, lower than both parents. a% denotes the percentage of HBP in DEG_FP_, b% denotes the percentage of CHP in DEG_FP_, c% denotes the percentage of B2P in DEG_FP_, d% denotes the percentage of CLP in DEG_FP_ and e% denotes the percentage of LBP in DEG_FP_. L1 and P1 represent flag leaves and anthers at meiosis stage, respectively. L2, P2, E2 and Z2 represent flag leaves, anther, embryo sac and leaf sheath at pre-flowering stage, respectively. L3, P3 and Z3 represent flag leaves, grain and leaf sheath at three days after flowering, respectively

The number of NAGs (non-additive genes) in each sample were ranged from 1013 to 3134, and accounted for 2.1–6.5% of total genes, and 44.1–60.2% of DEG_FP_, 57.3–72.4% of DEG_FPU_ and 17.8–51.1% DEG_C_ in all tissues (Table 7). GO analysis for NAG revealed that 102, 109, 110, 41, 34, 52, 37, 73 and 52 GO terms were significantly enriched in L1, L2, L3, P1, P2, P3, E2, Z2 and Z3, respectively. Interestingly, GO term of carbohydrate metabolic process was significantly enriched in all tissues except L3 (Additional file 13: Table S7).

**Table 7:** Non-additive expressed genes (NAGs) in F_1_ hybrid harboring double neutral genes

	Number of NAGs				Number of NAGs in DEGs					
	Down	Up	Total	%^a^	DEG_FP_	% ^b^	DEG_FPU_	% ^c^	DEG_C_	% ^d^
L1	1915	1739	3134	6.5	2980	56.8	1949	72.4	1031	40.4
L2	751	1739	2490	5.2	2356	56.5	1116	64.1	1240	51.1
L3	307	838	1145	2.4	1086	44.1	753	62.8	333	26.4
P1	1153	634	1787	3.7	1669	60.2	1556	67.2	113	24.7
P2	457	1226	1683	3.5	1549	49.1	929	61.8	620	37.5
P3	552	671	1223	2.5	1140	45.0	943	66.2	197	17.8
E2	271	742	1013	2.1	925	47.1	585	63.1	340	32.8
Z2	526	988	1514	3.2	1326	48.4	1039	63.7	287	25.9
Z3	955	1154	2109	4.4	1964	49.0	1435	57.3	529	35.2

Note: %^a^ denotes the percentage of NAGs in the total gene set (48,018), %^b^, %^c^, and %^d^ denote the percentages of NAGs in total number of DEG_FP_, DEG_FPU_ and DEG_C_, respectively. L1 and P1 represent flag leaves and anthers at meiosis stage, respectively. L2, P2, E2 and Z2 represent flag leaves, anther, embryo sac and leaf sheath at pre-flowering stage, respectively. L3, P3 and Z3 represent flag leaves, grain and leaf sheath at three days after flowering, respectively

### Mapping of DEG_FPU_ in known yield-related quantitative trait loci (QTLs)

**Fig. 5:**
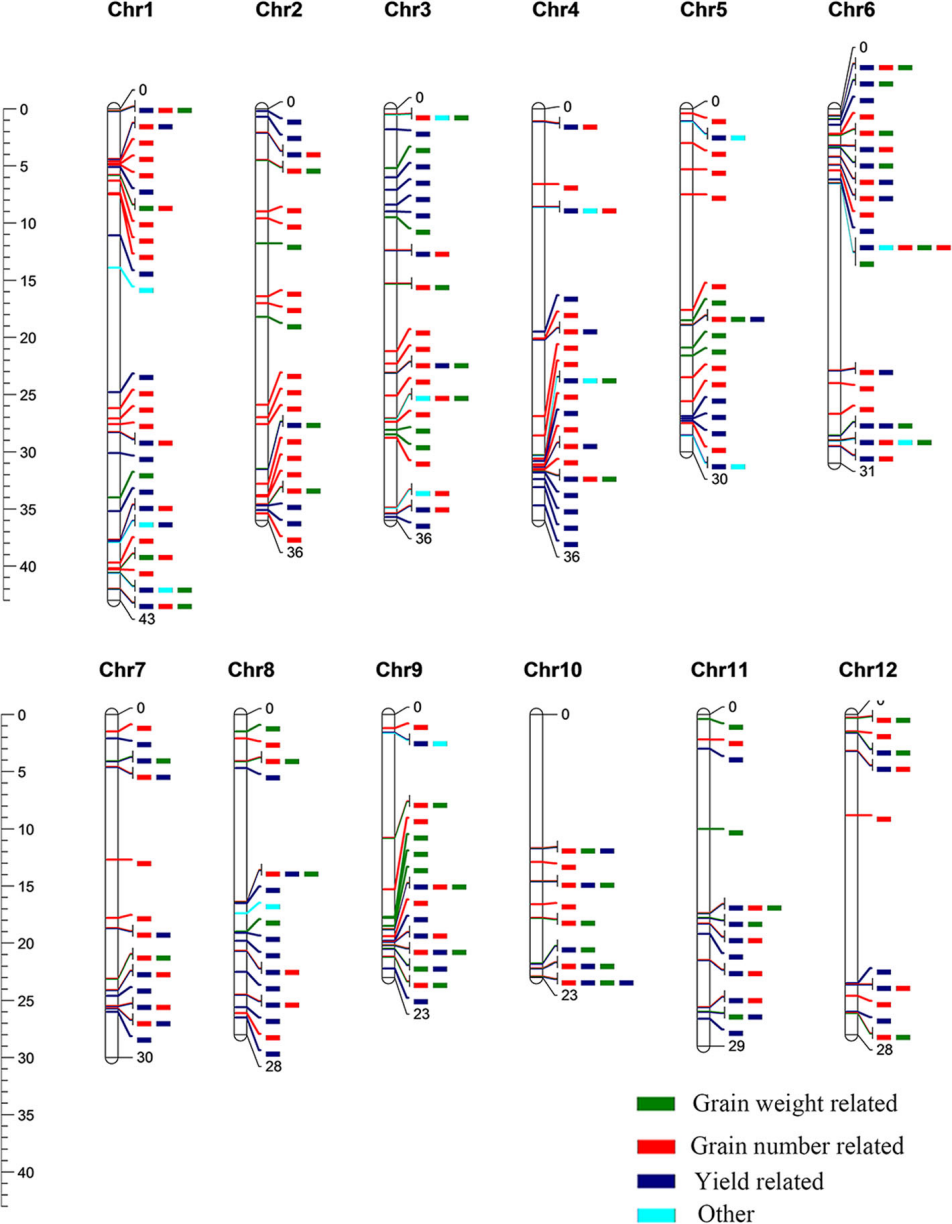
Distribution of DEG_FPU_ mapped onto yield-related QTLs. QTLs in Gramene (number of harbored genes ≤100) harboring DEG_FPU_ were aligned to the Michigan State University (MSU) Rice Genome Release 6.1

To understand the relationship between the observed DEG_FPU_ and autotetraploid hybrid yield-related traits, we explored DEG_FPU_ in yield related QTLs. We mapped all DEG_FPU_ onto 1019 yield-related QTLs and 26 traits present in the rice Gramene database. Among them, 2610, 1674, 1164, 2235, 1448, 1374, 896, 1585 and 2432 DEG_FPU_ were mapped onto yield-related QTLs in L1, L2, L3, P1, P2, P3, E2, Z2 and Z3, respectively, and more than 95% of the DEG_FPU_ were located in yield-related QTLs (Additional file 14: Figure S7 and Additional file 15: Figure S8). A total of 74, 221, 207, 267,199 and 42 QTL intervals contained 1~10, 11~50, 51~100, 101~ 200, 201~500 and more than 500 DEG_FPU_, respectively (Fig. 5 and S8B). The DEG_FPU_ were found in each yield-related QTLs expect 6 groups (Additional file 15: Figure S8C). Among DEG_FPU_-related QTLs, many QTLs are well characterized, including filled grain number (AQGH026, AQCB013, AQCF042, AQGH009, etc.), panicle number (AQCQ008, AQCE001, AQDQ015, AQFF055, etc.), grain yield per plant (AQCQ022, AQDQ048, AQED049, AQDQ018, etc.) and spikelet number (AQAB057, AQEF001, AQGP054, AQAI017, etc.). Functional annotations of some DEG_FPU_ can explain the potential association between DEG_FPU_ and QTL, for example relationship between *Starch synthase IIIa* (*LOC_Os08g09230*) and CQAS104 for yield, sucrose synthase (*LOC_Os03g22120*) and AQED032 for grain number, and invertase gene (*LOC_Os02g01590*) and AQFJ064 for grain yield per plant. The above listed results suggested that DEG_FPU_ can be correlated with heterosis in autotetraploid hybrid rice.

### Differentially expressed anther specific genes were associated with meiosis stage-specific genes in F_1_ compared to its parents

T449 is an autotetraploid rice line with low fertility and H1 is a neo-tetraploid rice line with high fertility, so we specifically investigated meiosis-specific genes associated with high fertility. We compared expression levels of genes between F_1_ vs T449 and H1 vs T449, and 381 genes were found to be commonly up-regulated between both comparisons in anthers during meiosis (Additional file 16: Table S8). GO analysis of these 381 genes showed that six biological process categories and nine molecular function categories were significantly enriched (Additional file 17: Table S9). We compared 381 up-regulated genes with microarray data of wild type rice anther meiosis stage-specific expression, and meiosis-related genes (Fujita et al. 2010; Tang et al. 2010; Deveshwar et al. 2011; Yant et al. 2013; Luo et al. 2014; Wright et al. 2015), and identified 4 meiosis-related genes including *LOC_Os09g32020* (*OsDFR*), *LOC_Os01g68870* (*MSP1*) and *LOC_Os12g24420* and *LOC_Os10g06770* (*CDKG1*), and 26 meiosis-specific expressed genes (Additional file 18: Table S10). Four pollen-related genes were identified from further analysis of 26 meiosis-specific expressed genes, including *LOC_Os03g07140 (DPW*), *LOC_Os04g24530* (*OsACOS12*), *LOC_Os06g40550* (*OsABCG15; PDA1*) and *LOC_Os08g03682* (*CYP703A3*).

**Fig. 6:**
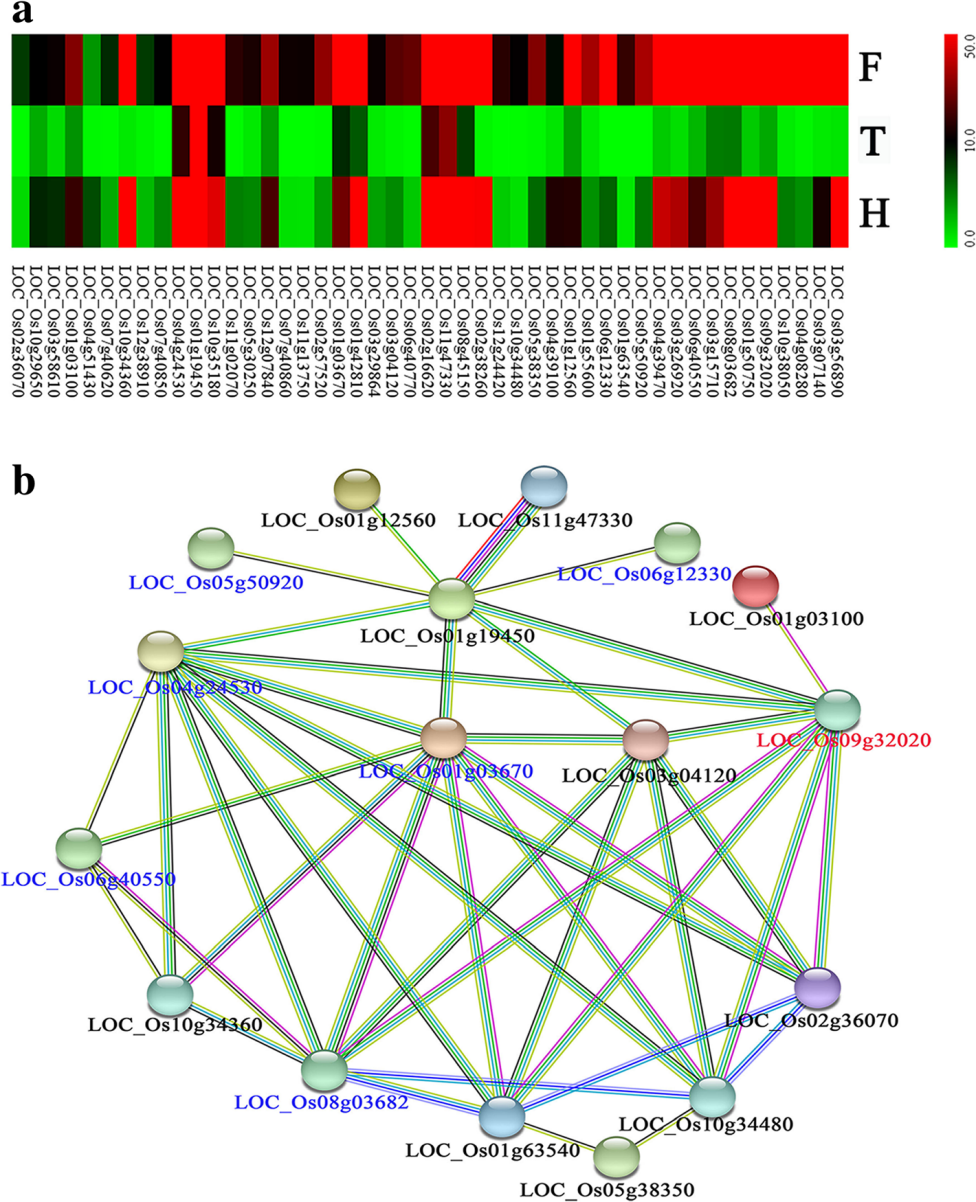
Gene expression levels of 47 genes and predicted protein-protein interaction network. **a**, The distribution of 47 genes exhibited up-regulation in F_1_ hybrid and H1 compared to T449 and down-regulation in T449 compared to E249 during meiosis stage, **b**, Predicted protein-protein interaction network of differently expressed genes (black), meiosis-specific (blue) and meiosis-related (red) genes. T represents T449, F represents hybrid, and H represents H1

Among 381 genes, 47 genes exhibited down-regulation in T449 compared to its diploid counterpart (E249) (Chen et al. 2018a) (Fig. 6a). Interestingly, two meiosis-related and 19 meiosis-specific genes were identified in these genes (Additional file 19: Table S11), including *LOC_Os09g32020* (*OsDFR*) and *LOC_Os12g24420* (*CDKG1*), and analysis of 19 meiosis-specific genes revealed four pollen-related genes, including *LOC_Os03g07140 (DPW*), *LOC_Os04g24530* (*OsACOS12*), *LOC_Os06g40550* (*OsABCG15; PDA1*) and *LOC_Os08g03682* (*CYP703A3*). We performed the predicted protein-protein interactions of 47 genes using STRING v10, and the results showed that 17 genes constituted genetic sub-networks, including one meiosis-related and six meiosis-stage specific genes. The meiosis-related gene (*OsDFR*, *LOC_Os09g32020*) interacted with two meiosis-specific gene, including *LOC_Os08g03682* (*CYP703A3*), encodes cytochrome P450 hydroxylase and *LOC_Os04g24530* (*OsACOS12*), encodes acyl-CoA synthetase 12, which interacted with six differently expressed genes, including three cytochrome P450, two AMP-binding enzyme and a protein binding protein (Fig. 6b).

### Co-expression network analysis of differentially expressed genes in different tissues by weighted gene co-expression network analysis (WGCNA)

**Fig. 7:**
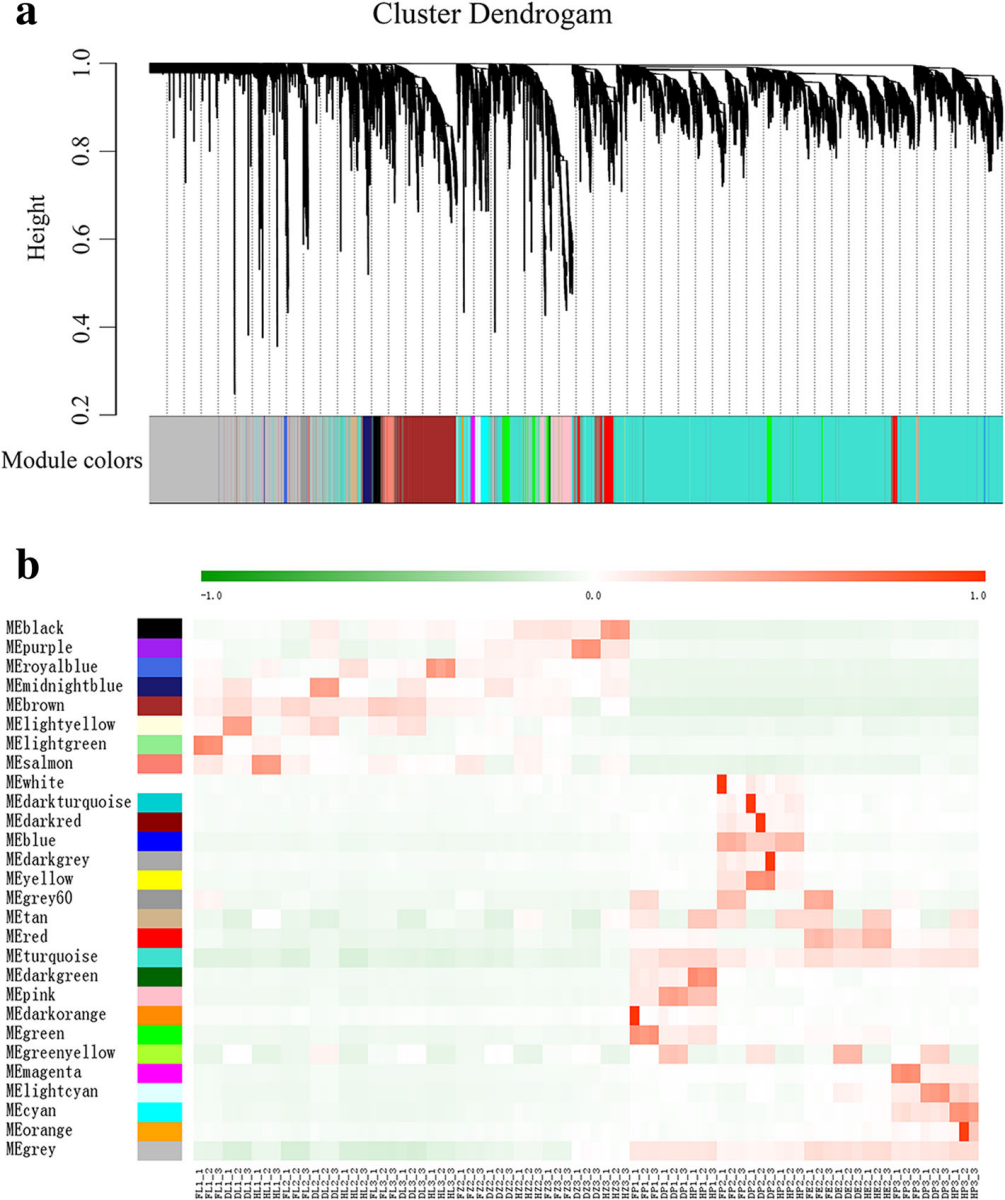
WGCNA based gene expression matrix between hybrid and parents. **a** Hierarchical cluster tree showing co-expression modules identified by WGCNA. Each leaf in the tree represents one gene. The major tree branches constitute 28 modules labeled with different colors. **b** Module-sample relationship. Each row corresponds to a module. Each column corresponds to a sample. The color of each cell at the row-column intersection indicates the correlation coefficient between the module and the sample

WGCNA, which is a systems biology tool, was used to understand the relationships and networks in a set of genes. In this study, WGCNA was constructed using RNA-seq data, and 28 WGCNA modules were identified (Fig. 7a, Additional file 20: Table S12). The gene numbers in these modules were ranged from 30 (MEwhite module) to 9294 (MEgrey module). Interestingly, the turquoise and grey modules consist of 67.72% of the genes in the network analysis. We found that some modules showed correlation with the different tissues in F_1_ and parents (Fig. 7b), for example, MEbrown module in leaf, MEblue module in mature anther, MEred module in mature embryo sac, and MEturquoise and MEgrey module in three tissues (anther, embryo sac and grain), which indicated that these modules may play putatively important roles in tetraploid rice leaf and reproductive organs. Furthermore, a total of 1335 genes were involved in the MEbrown module, and GO enrichment analysis showed significant enriched terms that were related to photosynthesis light harvesting, light reaction, carboxylic acid metabolic process and chlorophyll metabolic process. These results indicated that brown module genes may play an important role in the photosynthesis in tetraploid rice. In total, 9291 genes were involved in the MEturquoise module, and GO analysis revealed significant terms associated with DNA repair, carbohydrate metabolic process and transport, which indicated that MEturquoise module plays an important role in the fertility and yield of tetraploid rice (Additional file 21: Table S13).

### Association between DNA sequence variations and differentially expressed genes in F_1_ hybrid compared to parents

A total of 100,395,344 and 150,936,032 clean reads were obtained in T449 and H1 by using genome re-sequencing, respectively. Approximately 98.16% (T449) and 96.11% (H1) of clean reads were mapped onto the Nipponbare reference genome, and the reads coverage depths were 35× and 49× in T449 and H1, respectively (Additional file 22: Table S14). A total of 912,892 SNPs and 195,976 InDels were detected between T449 and H1 by using the two filter conditions (coverage ≥10 and ≤ 100, and removal of heterozygous SNPs and InDels). We found that about 5% of SNPs and 6% of InDels were detected in intergenic regions, and 60% SNPs and 65% InDels were identified in up or down regulatory regions, which might be related to the differentially expressed genes (Additional file 23: Table S15). Furthermore, we identified the genes variations (SNP + InDel) in the DEG_FPU_, and found that 2044, 1297, 906, 1777, 1132, 1053, 707, 1232 and 1880 genes displayed variations between T449 and H1 in L1, L2, L3, P1, P2, P3, E2, Z2 and Z3, respectively (Additional file 24: Table S16). Of these genes, 53, 71, 79, 27, 3, 24, 18, 56 and 49 GO groups were significantly enriched in the L1, L2, L3, P1, P2, P3, E2, Z2 and Z3, respectively. Interestingly, GO term of carbohydrate metabolic process was significantly enriched in all tissues except L3 (Additional file 25: Table S17). These results were nearly consistent with the GO enrichment of DEG_FPU_.

### Expression patterns of saccharides metabolism and starch synthase related genes in the hybrid compared to parents

**Fig. 8:**
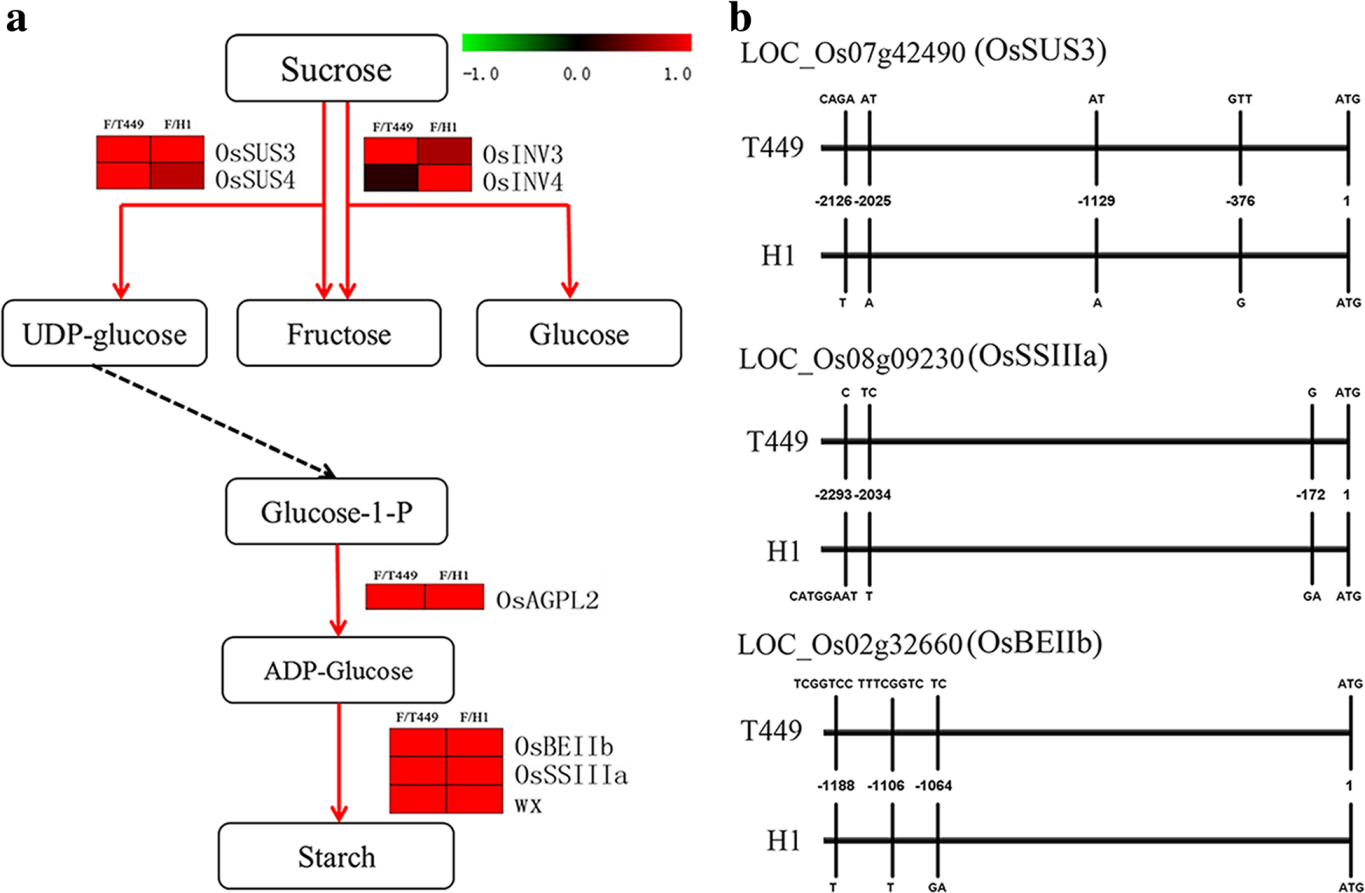
Predicted carbohydrate pathways in grains three days after flowering and related-gene variations in parents. **a** Starch pathway in grains of polyploid rice. The log2-transformed ratio between hybrid and parents was drawn by heatmap (F, F_1_ hybrid; T449, maternal line; H1, paternal line). **b** Sequence comparisons of *OsSUS3, OsBEIIb* and *OsSSIIIa* in T449 and H1

The GO and KEGG analyses of DEG_FPU_ showed that there were significant differences for carbohydrate metabolic process in nine tissues between F_1_ and its parents, and DEG_FPU_ were involved in sucrose synthase, cell wall invertase, 6-phosphofructokinase, and hexokinase. Many saccharide metabolic genes were up-regulated in the F_1_ compared to its parents in L1, P1 and P3 (Additional file 26: Table S18). Interestingly, the saccharide transporters were up-regulated in the F_1_ compared to its parents in L1, P1 and P3, and these results were consistent with saccharide metabolic genes (Additional file 26: Table S18). The two saccharide transporter genes (*LOC_Os02g10800* and *LOC_Os03g07480*) were also up-regulated in F_1_ compared to both parents in P3 (Additional file 26: Table S18). In addition, the invertase (*OsINV3* and *OsINV4*), sucrose synthase (*OsSUS3* and *OsSUS4*), hexokinase gene (*OsHXK6*), starch branching enzyme (*OsBEIIb*) and two starch synthase genes (*OsSSIIIa* and *wx*) displayed higher levels of expressions in F_1_ than parents in the grains (three days after flowering) (Fig. 8a). Moreover, the promoter regions of *OsSUS3, OsBEIIb* and *OsSSIIIa* also exhibited differences between maternal and paternal rice lines by re-sequencing (Fig. 8b).

## Discussion

### High heterosis and the frequency of bivalents in F_1_ hybrid harboring double neutral genes

In the current study, the paternal line was high fertility neo-tetraploid rice and maternal line was autotetraploid rice harboring double neutral genes. The hybrid displayed stronger heterosis for yield and yield-related traits, such as filled grains per plant, total grains per plant, grain yield per plant and seed setting. These results were consistent with the previous studies, where autotetraploid hybrids exhibited high heterosis for filled grains per panicle, grain yield per plant and seed setting (Shahid et al. 2012; Guo et al. 2017). In addition, the pollen and embryo sac fertilities were investigated to evaluate the fertility of hybrid and its parents. Our results showed that the embryo sac fertility of hybrid and its parents was higher than 89%, and pollen fertility of hybrid and paternal line was high, while pollen fertility of maternal line was low. These results revealed that pollen fertility has a greater impact on seed setting than embryo sac fertility in autotetraploid rice hybrid and its parents.

It is well known that meiosis process has a great effect on plant reproductive development, and chromosome behavior and configuration play an important role in the plant meiosis and directly correlated with pollen fertility. The way of quadrivalent separation depends on chromosome configuration. Many quadrivalent chromosomes were found in *Triticum monococcum* during diakinesis and metaphase I (Kim and Kuspira 1993), while chain, ring and frying pan shapes were three main types of chromosome configurations in autotetraploid rice (Luan et al. 2007; He et al. 2011a). Ring, chain, frying pan, “X” and “OK” shapes were observed in the present study, and we have drawn these shape models based on the observation. The ring shape quadrivalent was found more frequently compared to other quadrivalent types in the present study. Our results were in agreement with the previous studies, who also observed ring shape quadrivalent in autotetraploid rice (Luan et al. 2007; He et al. 2011b). He et al. (2011b) revealed that high frequency of bivalent was related to high pollen fertility and seed setting in autotetraploid rice hybrid. We also observed higher frequency of bivalents in the autotetraploid hybrid than parents at diakinesis and metaphase I. Chromosome behavior had a direct relationship with pollen fertility and seed setting in autotetraploid rice hybrids and interspecific hybrid between *Brachiaria ruziziensis* and *B. brizantha* (Adamowski et al. 2008; He et al. 2011b; Guo et al. 2016). Here, the frequency of normal chromosome behavior was significantly higher in the hybrid with high fertility than maternal line with double neutral genes.

It is worth to mention that the frequency of abnormal chromosome behavior is much higher during anaphase II than other stages in this study. Here, many types of abnormal chromosome behaviors, including asynchronous meiocytes, abnormal spindles and straggling chromosomes were observed at anaphase II (Additional file 5: Table S3). Of these abnormalities, asynchronous meiocytes was the highest, and the frequencies of asynchronous meiocytes were 66.9%, 65.3% and 56.0% in T449, F_1_ and H1, respectively. We inferred that most of asynchronous meiocytes could develop normal tetrad according to their morphology, which might be the major reason for lower frequency of the normal cells at anaphase II than other phases.

### The expression patterns of meiosis and meiosis-related genes promote high fertility in F_1_ hybrid

A number of meiosis-related and meiosis-specific genes were detected in rice (Fujita et al. 2010; Tang et al. 2010; Deveshwar et al. 2011; Yant et al. 2013; Luo et al. 2014; Wright et al. 2015). A total of 55 meiosis-related or meiosis-stage-specific genes were found to be down-regulated, which increased pollen sterility loci interactions in autotetraploid rice hybrids (Wu et al. 2015). In the present study, the meiotic stages were determined by the floret length and according to the observation by 4′, 6-diamidino-2-phenylindole (DAPI) staining (He et al. 2011a). The DAPI staining could clearly distinguish between meiotic stages and other pollen development stages. For the proper understanding of meiosis-related and meiosis-specific genes between hybrid and parents, we dissected anthers from floret for RNA-seq analysis. A total of four meiosis-related and 26 meiosis-stage specific genes were identified, which were found to be up-regulated in hybrid and paternal line compared to maternal line. Interestingly, of the four meiosis-related and 26 meiosis-specific genes, two meiosis-related and 19 meiosis-specific genes were also found to be down-regulated in autotetraploid rice compared to diploid progenitors (Chen et al. 2018a). For example, *DPW* gene encodes a fatty acyl ACP reductase, and was found to be essential for anther cuticle, pollen wall and pollen sporopollenin biosynthesis (Shi et al. 2011), and maternal and paternal lines showed upstream and intron variations in *DPW* gene. *OsACOS12*, which is an acyl-CoA synthetase, is essential for sporopollenin synthesis in rice (Li et al. 2016a; Yang et al. 2017), and maternal and paternal lines displayed downstream, upstream, intron and non-synonymous variations in *OsACOS12*. *PDA1* encodes an ABC transporter (*OsABCG15*) and required for the transport of lipidic precursors for anther cuticle and pollen exine development (Zhao et al. 2015). *CYP703A3*, cytochrome P450 hydroxylase, is involved in the tapetum degeneration retardation, a known pollen exine formation (Yang et al. 2014). The meiosis-related gene (*LOC_Os12g24420*) encoded cyclin-dependent kinase, which is homolog to *CDGK1* in *Arabidopsis*, and *CDKG1* protein kinase is crucial for synapsis and recombination in *Arabidopsis* during meiosis (Zheng et al. 2014), and we observed changes in the downstream and intron region of *CDKG1* in maternal and paternal lines*.* These results suggested that the expression profiles of important meiosis-related or meiosis-specific genes have a significant effect on the fertility of polyploidy hybrid rice.

### Dominance, non-additive and yield-related genes/QTLs contribute to heterosis

The DEG_FP_ were divided into five basic groups according to their expression profiles, including over-dominance (HBP), under-dominance (LBP), dominance (CHP and CLP), and mid-parent (BBP) (Liu et al. 2018). In our data, the dominance expression was the most prevalent class among DEG_FP_ (46.34–77.71%). Similarly, the dominance expression patterns were found to be the most abundant among DEG_FP_ in wheat and rice hybrids by RNA-seq analysis (Zhai et al. 2013; Liu et al. 2018). These results indicated that dominance expression have a great effect on the performance of hybrids. According to gene expression levels of hybrid and its parents, the gene expression profiles of F_1_ could be divided into two types, the first type is called as additive expression, which is contributed by each allele from its parents in a hybrid, and another is non-additive expression that differed from the mid-parent value (MPV) (Wei et al. 2009). In the previous study, whether or not a transcript shows non-additive expression is most likely to be affected by the contributions of *cis-* and *trans*-acting element of a gene (Zhang et al. 2008). In this study, NAGs only accounted for 2.1–6.5% of the total expressed genes, but 57.3–72.4% of DEG_FPU_ were NAGs in each tissue. These results were consistent with diploid rice (Wei et al. 2009), wheat (Liu et al. 2018) and maize heterosis (Swanson-Wagner et al. 2006), who also detected NAGs in F_1_. The previous studies have shown that NAGs play vital roles in heterosis (Zhang et al. 2008; Liu et al. 2018), and NAGs were associated with circadian rhythm, flowering time, and panicle branching in rice (Li et al. 2016b). Overall less number of NAGs detected in this study, but major portion of DEG_FPU_ was constituted of NAGs. Therefore, we speculated that NAGs play important roles in high F_1_ heterosis of polyploid rice.

The potential relationships between differently expressed genes and QTLs have been proposed in many yield-related QTL regions using RNA-seq (Zhai et al. 2013; Chen et al. 2018b). A recent study showed that nine genes (*Hd3a*, *TAC1*, *Ghd8*, *Sd-1*, *NAL1*, *Hd1*, *GW6a*, *IPA1* and *DEP1*) have a major impact on heterosis in diploid hybrid rice (Huang et al. 2016). In our study, *DEP1*, which is related to rice panicle and located in SKPNB (spikelet number, AQBK037), was found to be up-regulated in grains (three days after flowering) of F_1_ hybrid compared to maternal line and also up-regulated in leaf of hybrid than parents during meiosis stage. *GW6a*, which involved in grain-weight and located in TSDWT (1000-seed weight, AQEB012), revealed much higher expression in anthers of hybrid than parents during meiosis. The semi-dwarf gene (*Sd-1*), which is involved in biosynthesis of gibberellin and located in GRYLD (grain yield, AQQ005), exhibited higher levels of expressions in leaf sheath (three day after flowering) of hybrid than parents. *Hd3a*, which is related to rice flowering and regional adaptation, and located in FGRNB (filled grain number, AQCF008), was up-regulated in leaf (before flowering) of hybrid compared to parents. These results showed that these candidate genes in QTL regions may contribute to heterosis in autotetraploid hybrid rice.

### Saccharides metabolism and starch synthase related genes play an important role in heterosis

Carbohydrate metabolism plays an essential role in the plant growth and development. RNA-seq analysis showed that processes of carbohydrate metabolism are related to heterosis in rice (Wei et al. 2009; Zhai et al. 2013). In addition, our research group reported that abnormal distribution of saccharides and saccharides-related genes may influence pollen fertility and cause decrease in the yield of autotetraploid rice (Chen et al. 2018a). Here, the DEG_FPU_ was significantly enriched in the carbohydrate metabolism process between hybrid and its parents in nine tissues sampled across different development stages. The grain filling stage is an important part of growth and development in rice, and a large quantity of carbohydrates are synthesized and transported into the grain at this stage, particularly the embryo starts exponential growth at three days after flowering that shows dual rhythmicity (Itoh et al. 2005). The stage of three days after flowering is important stage for grain development, so we focused on saccharides metabolism in the grains three days after flowering. The carbohydrates supply from the leaves to pollen or grain involves sucrose transport and degradation, monosaccharides formation and transport, and starch generation (Ruan 2012). There are two types of enzymes that catalyze the sucrose degradation in plants, one is sucrose synthase (SUS) and the other is invertase (Ruan et al. 2010). Here, the invertase (*OsINV3* and *OsINV4*) and sucrose synthase (*OsSUS3* and *OsSUS4*) were found to be up-regulated in the grains of hybrid compared to parents. After sucrose degradation, the resulting hexoses undergo phosphorylation by hexokinase for starch synthesis. Subsequently, hexokinase plays important role in hexose signaling and sensing (Cho et al. 2009; Kim et al. 2016). The hexokinase gene (*OsHXK6*) was up-regulated in grains (three after days flowering) of the hybrid compared to its parents. The starch synthase, starch debranching and starch branching enzyme have a great influence on the starch generation and metabolism (Zeeman et al. 2010). The starch branching enzyme (*OsBEIIb*) and two starch synthase (*OsSSIIIa* and *wx*) genes were up-regulated in the grains of hybrid compared to parents. In addition, we detected differences between maternal and paternal rice lines in the promoter regions of *OsSUS3, OsBEIIb* and *OsSSIIIa* by re-sequencing. Consequently, the genetic effects of *OsSUS3, OsBEIIb* and *OsSSIIIa* may cause allelic heterozygosity in promoter regions of hybrid.

Sucrose and monosaccharide transporters are important proteins for the translocation of saccharides from source to sink organs (Ruan et al. 2010). *OsSUT1* primarily play a role in the transport pathway (Scofield et al. 2007). In our study, sucrose transporter (*OsSUT1*) displayed much higher expression patterns in the grain (three after days flowering) of hybrid than parents. *OsBT1*, which encodes putatively ADP-glucose transporter and localizes in the amyloplast envelope membrane, plays a crucial role in starch synthesis (Cakir et al. 2016; Li et al. 2017a). We found that *OsBT1* was up-regulated in the grain (three after days flowering) of hybrid compared to parents. Transcriptome profiling showed high expression levels of saccharides metabolism and starch synthase related genes in the hybrid, which might be an indication of enhanced source for sink tissues and resulted in high yield of hybrid.

In our previous studies, we found that the double neutral genes can overcome the hybrid sterility in autotetraploid rice (Wu et al. 2017), and detected specific differentially expressed genes associated with fertility and heterosis in neo-tetraploid rice by RNA-seq (Guo et al. 2017). Here, an autotetraploid rice line (T449), harboring *Sa-n* and *Sb-n* double neutral genes for pollen sterility loci, was used to generate the hybrids by crossing with neo-tetraploid rice, and investigated the heterosis and fertility by cytological and RNA-seq methods. We further want to understand the role of double neutral genes in heterosis and fertility of neo-tetraploid rice. Therefore, we observed chromosome behavior and gene expression patterns during important growth stages. The results showed that seed setting of F_1_ hybrid improved with the increase in number of bivalents, and many important genes, including meiosis-related and meiosis-specific genes and saccharides metabolism and starch synthase related genes, exhibited heterosis specific expression patterns in polyploid rice during different development stages.

## Conclusions

In this study, we observed the chromosome behavior and configuration in hybrid and its parents, and found higher frequency of bivalent and normal chromosome behavior in hybrid than parents, which promoted high fertility (heterosis) in the hybrid harboring double neutral genes. Furthermore, we systematically investigated the global transcriptome of hybrid and its parents by RNA-seq. We obtained a large number of DEG_FPU_, and detected substantial candidate genes, including meiosis-related and meiosis-specific genes, saccharides metabolism and starch synthase related genes, which were up-regulated in hybrid having improved fertility and yield. Our results provided new resource for polyploid rice breeding and exploring of these candidate genes will provide valuable information for revealing molecular mechanisms of heterosis in polyploidy rice.

## Methods

### Rice material

An autotetraploid rice line, DN18-4x (T449), harboring *Sa-n* and *Sb-n* double neutral genes for pollen sterility loci, was used to generate the hybrids by crossing with five neo-tetraploid rice lines, including Huaduo 1 (H1), Huaduo 2 (H2), Huaduo 3 (H3), Huaduo 4 (H4) and Huaduo 8 (H8). All the materials were planted at the experimental farm of South China Agricultural University (SCAU) under natural conditions, and management practices followed the recommendations for the area.

### Investigation of agronomic traits and data analysis

Agronomic traits, including plant height, effective number of panicles per plant, grain length and width, 1000-grain weight, filled grains per plant, total grains per plant, grain yield per plant and seed setting, were investigated. The standard for investigating these agronomic traits was according to the protocols of People’s Republic of China for the registration of a new plant variety DUS (Distinctness, Uniformity and Stability) test guidelines of rice (Guidelines for the DUS test in China, 2012) (Guo and Liu 2014; Guo et al. 2017). The mid-parent heterosis (MPH) and high-parent heterosis (HPH) were estimated by the following formula: MPH = (F_1_ − MP)/MP × 100%, and HPH = (F_1_ − HP)/HP × 100%, where F_1_ related to the performance of hybrid, HP was defined as the performance of the best parent, and MP was defined as an average performance of two parents (Guo and Liu 2014).

### Cytological observation

The chromosome configuration and behaviors were observed according to Wu et al. (2014). The inflorescences of F_1_ and its parents lines were collected from the shoots of rice plants with 0 to 4 cm between their flag leaf cushion and the second to last leaf cushion, and fixed in Carnoy’s solution (ethanol: acetic acid = 3:1) for 24 h, and the samples were stored in 70% ethanol at 4 °C after washing two times. The anther was removed from the floret and placed in a small drop of 1% acetocarmine on a glass slide. The glass slide was covered with a slide cover after 2–3 min, and observed under a microscope (Motic BA200).

The pollen fertility was observed according to Shahid et al. (2013b). The normal and abnormal pollens were observed by staining with 1% I_2_-KI under a microscope (Motic BA200). The whole mount eosin B confocal laser scanning microscopy (WE-CLSM) was used to investigate the embryo sac fertility in F_1_ and its parents according to Li et al. (2017b) with minor modifications. The ovary was dissected from the floret, and was hydrated in 70%, 50%, 30%, 10% ethanol and distilled water for 30 min each. Then, the samples were dehydrated in 10%, 30%, 50%, 70%, 90% and 100% ethanol for 30 min after eosin B staining for 12 h. Finally, the samples were shifted into a mixture solution (ethanol and methyl salicylate = 1:1) for 2 h, and then keep in pure methyl salicylate and observed under a laser scanning confocal microscope (Leica SPE).

### RNA-seq experiments and data analysis

All samples were collected during meiosis, pre-flowering, and three days after flowering. The meiosis stage is a crucial event for the sexual reproduction of eukaryotes to form haploid spores and gametes (Luo et al. 2014). The pre-flowering stage is an important stage for pollen and embryo sac fertility. The carbohydrates are synthesized and transported into the grains in large quantity during grain filling stage (Itoh et al. 2005). The nutrients produced by leaves are transported to other organs through the leaf sheath, so the leaf sheath has an important role in the transportation of energy. Flag leaf is one of the most important photosynthetic organs in rice and has an important impact on crop yield and quality. In addition, pollen and embryo sac have a significant impact on rice fertility and yield (Shahid et al. 2013b; Wu et al. 2015; Li et al. 2017b). Hence, we collected the nine tissues during these development stages from hybrid and its parents in three biological replicates, including anthers (P1) and flag leaves (L1) at meiosis stage, and flag leaves (L2), leaf sheath (Z2), anther (P2) and embryo sac (E2) at pre-flowering stage, and flag leaves (L3), leaf sheath (Z3) and grains (P3) at three days after flowering (Additional file 4: Figure S2).

All tissues of hybrid and its parents were harvested in three biological replicates and immediately kept at − 80 °C for RNA extraction. The total RNA was extracted according to the manual instructions of the TRIzol Reagent (Life technologies, California, USA). The library was prepared according to the vendor’s recommended protocol. The RNA-seq was performed on the Illumina HiSeq 4000 sequencing platform (LC Sceiences, USA). Using the Illumina paired-end RNA-seq approach, we sequenced the transcriptome that generated millions of paired-end reads. Low quality reads, including reads containing sequencing adaptors, reads containing sequencing primer and nucleotides with quality score lower than 20, were removed. The mapped reads from each sample were assembled using StringTie, and all transcriptome samples were mixed to reconstruct a comprehensive transcriptome by perl scripts. After the generation of transcriptome, the StringTie and Ballgown were used to evaluate the gene expression levels. StringTie was used to perform expression level for mRNAs by calculating FPKM (fragments per kilobase of transcript per million fragments mapped reads), and false discovery rate (FDR) was used to determine the threshold of the *P*-value in multiple tests.

The Venny software was used to identify the overlapped differentially expressed genes in different samples (http://bioinfogp.cnb.csic.es/tools/venny/index.html). Hierarchical analysis was carried out for all genes using Cluster 3.0 software after normalization. Transcription factor analysis was done according to transcription factor data (Jin et al. 2017). Gene Ontology (GO) enrichment analysis was employed for functional categorization by using AgriGO tool (http://systemsbiology.cau.edu.cn/agriGOv2/).

### Expression patterns of differentially expressed genes (DEGs)

The expression patterns of DEGs were defined according to Liu et al. (2018). We defined the gene expression in F_1_ as EF_1_, and genes expression of both parental lines as ET449 and EH1.We defined the average value of both parental lines as MPV (mid-parental value). If the F_1_ was significantly (FDR ≤ 0.05 and fold change ≥2) different from MPV, we defined these genes as non-additive genes (NAGs), if there was non-significant difference between F_1_ and MPV, these genes were defined as additive genes. Classification of DEG_FP_ was performed according to the expression of EF1 relative to ET449 and EH1. “>” and “<” represents statistically higher or lower, and “=” represents statistically similar. If EF1 > ET449 > EH1, or EF1 > EH1 > ET449, then expression patterns of these genes were considered as higher than both parents (HBP); if EF1 = ET449 > EH1, or EF1 = EH1 > ET449, then expression patterns of these genes were considered as close to higher parent (CHP); if ET449 > EF1 > EH1, or EH1 > EF1 > ET449, then expression patterns of these genes were considered as between two parents (BBP); if EF1 = ET449 < EH1, or EF1 = EH1 < ET449, then expression patterns of these genes were considered as close to lower parent (CLP); if EF1 < ET449 = EH1, or EF1 < ET449 < EH1, or EF1 < EH1 < ET449, these genes were considered as lower than both parents (LBP).

### Real-time qRT-PCR analysis

A total of 12 DEGs were randomly selected for validation of RNA-Seq data by qRT-PCR. The gene-specific primers were designed using Primer Premier 5.0 software, and checked in the NCBI (National Center for Biotechnology Information) for specific primers (Additional file 27: Table S19). Total RNA was taken from sequenced samples, and the first-strand cDNA was synthesized using the Transcriptor cDNA Synth. Kit 1 (Roche) according to the manufacturer’s instructions. The qRT-PCR reaction procedure was 30 s at 95 °C, with 40 cycles of 95 °C denaturation for 10 s and 60 °C annealing and extension for 30 s, and performed on the Lightcycler480 system (Roche). The genes relative expression levels were calculated using the 2-^ΔΔCt^ method (Livak & Schmittgen 2001). All qRT-PCR reactions were performed in triplicate.

### Mapping DEG_FPU_ to rice QTLs and weighted gene co-expression network analysis (WGCNA)

Rice QTL data with physical positions on the MSU Rice Genome Annotation Project Release 6.1 were acquired from Gramene (Youens-Clark et al. 2011). The DEG_FPU_ were mapped onto 1019 yield related QTLs and 26 yield-related traits using gene coordinates from the MSU Rice Genome Annotation Project. The gene co-expression networks were used WGCNA package in R (Langfelder & Horvath 2008). To reduce noise, genes with total FPKM < 5 in 81 samples were removed. The modules were obtained using the automatic network construction with default settings.

### Whole-genome re-sequencing analysis

The young leaves of T449 and H1 were collected and genomic DNA was extracted using a modified CTAB method (Cota-Sanchez et al. 2006). The process of genomic re-sequencing was performed on the Illumina Hiseq 2500 platform (Biomarker Technologies, Beijing, China). The procedure was performed according to the standard Illumina protocol (Bai et al. 2013). The generated FASTQ file quality was evaluated using FastQC (http://www.bioinformatics.babraham.ac.uk/projects/fastqc/). The three filter conditions (reads with sequencing adapter, reads with more than 10% N content, reads with more than 50% low quality bases (< 10) were used to remove low-quality reads, and then the high quality reads were mapped onto the Nipponbare reference genome using BWA software. The GATK software was used to identify SNPs and InDels, and the SnpEff software was used to annotate the SNPs and InDels based on the Nipponbare reference genome.

## Additional files

Additional file 1: **Table S1.** Heterosis analysis of hybrids generated by the crossing of T449 and neo-tetraploid rice lines. (DOCX 17 kb)

Additional file 2: **Table S2.** Embryo sac fertility of hybrid and parents. (DOCX 15 kb)

Additional file 3: **Figure S1.** Chromosome behavior of hybrid. (PPTX 504 kb)

Additional file 4: **Figure S2.** Collection of plant tissues for RNA-Seq during different development stages. (PPTX 79 kb)

Additional file 5: **Table S3.** Frequency of cells exhibiting abnormal chromosome behavior in pollen mother cells (PMC) during meiosis. (XLSX 11 kb)

Additional file 6: **Table S4.** Quality of RNA sequencing data and information of reads aligned to the Nipponbare reference genome. (XLSX 17 kb)

Additional file 7: **Table S5.** Correlation analysis between all tissues. (XLSX 96 kb)

Additional file 8: **Figure S3.** The principal component analysis (PCA) in the hybrid and its parents. (PPTX 199 kb)

Additional file 9: **Figure S4.** Hierarchical clustering analysis of all gene models based on expression data. (PPTX 557 kb)

Additional file 10: **Figure S5.** Comparison of the log2 (FC) of 12 selected transcripts using RNA-Seq and qRT-PCR. (PPTX 90 kb)

Additional file 11: **Figure S6.** The number of DEG_FPU_ belonging to different transcription factor families detected in the hybrid and its parents. (PPTX 222 kb)

Additional file 12: **Table S6.** Gene ontology (GO) enrichment analysis for DEG_FPU_ in nine tissues. (XLSX 39 kb)

Additional file 13: **Table S7.** Gene ontology (GO) enrichment analysis for NAGs in nine tissues. (XLSX 49 kb)

Additional file 14: **Figure S7.** Distribution of DEG_FPU_ mapped in yield and yield-related QTLs. (PPTX 1011 kb)

Additional file 15; **Figure S8.** Distribution of DEG_FPU_ on yield-related QTLs. (PPTX 241 kb)

Additional file 16: **Table S8.** Gene IDs of common up-regulated genes between F_1_ vs T449 and H1 vs T449 in anther at meiosis stage. (XLSX 14 kb)

Additional file 17: **Table S9.** GO analysis of common up-regulated genes between F_1_ vs T449 and H1 vs T449 in anther at meiosis stage. (DOCX 17 kb)

Additional file 18: **Table S10.** Meiosis related and stage-specific genes detected in anther during meiosis. (XLSX 11 kb)

Additional file 19: **Table S11.** Up-regulated DEGs-sp in F_1_ compared to T449 were found to be overlapped with the down-regulated genes in T449 compared to E249 (diploid). (XLSX 11 kb)

Additional file 20: **Table S12.** List of WGCNA module genes. (XLSX 465 kb)

Additional file 21: **Table S13.** Gene ontology (GO) enrichment analysis of WGCNA module genes. (XLSX 76 kb)

Additional file 22: **Table S14.** Summary of general sequencing data of maternal line (T449) and paternal line (H1) mapped onto Nipponbare reference genome. (DOCX 16 kb)

Additional file 23: **Table S15.** Effect type annotation and distribution of SNPs and InDels in different genomic regions. (DOCX 18 kb)

Additional file 24: **Table S16**. Gene IDs of DEG_FPU_ combined with variations between parents lines in nine tissues. (XLSX 153 kb)

Additional file 25: **Table S17.** Gene ontology (GO) enrichment analysis for DEG_FPU_ combined with gene variations between parental lines in the nine tissues. (XLSX 33 kb)

Additional file 26: **Table S18.** The DEG_FPU_ involved in saccharide transporter and metabolism in the hybrid compared to parents. (XLSX 13 kb)

Additional file 27: **Table S19.** List of primers used for qRT-PCR. (DOCX 16 kb)
