# Supplementary material for: Carbohydrate metabolism and fertility related genes high expression levels promote heterosis in autotetraploid rice harboring double neutral genes
Source: Rice (N Y). 2019 May 10;12:34. doi: 10.1186/s12284-019-0294-x (PMC6510787; doi:10.1186/s12284-019-0294-x)
Supplement: Supplementary file 1 — Table S1. Heterosis analysis of hybrids generated by the crossing of T449 and neo-tetraploid rice lines. (DOCX 17 kb) [file 12284_2019_294_MOESM1_ESM.docx]

**Table S1.** Heterosis analysis of hybrids generated by the crossing of T449 and neo-tetraploid rice lines

| Traits | Mid parent heterosis | | | | |  | High parent heterosis | | | | |
| --- | --- | --- | --- | --- | --- | --- | --- | --- | --- | --- | --- |
|  | Average value (%)(±SD) | Variable coefficient | Variation range | + | - |  | Average value (%) | Variable coefficient | Variation range | + | - |
| PH | 21.14 | 0.12 | 17.56~24.44 | 5 | 0 |  | 1.69 | 4.05 | -3.99~13.6 | 2 | 3 |
| EP | 21.47 | 0.55 | 8~33.33 | 5 | 0 |  | 9.84 | 1.74 | -13.16~25 | 3 | 2 |
| GL | 2 | 1.37 | -0.68~5.86 | 3 | 2 |  | -5.17 | -0.76 | -8.72~-0.54 | 0 | 5 |
| GW | 4.29 | 0.73 | 1.16~8.54 | 5 | 0 |  | -2.54 | -1.89 | -7.88~4.68 | 1 | 4 |
| TGW | 7.31 | 0.54 | 3.63~13.35 | 5 | 0 |  | -2.26 | -3.4 | -9.49~9.96 | 1 | 4 |
| FGP | 163.44 | 0.36 | 66.61~226.6 | 5 | 0 |  | 71.1 | 0.53 | 6.49~106.74 | 5 | 0 |
| TGP | 37.78 | 0.63 | -0.97~56.94 | 4 | 1 |  | 34.65 | 0.65 | -1.61~52.54 | 4 | 1 |
| GYP | 170.89 | 0.19 | 119.28~205.41 | 5 | 0 |  | 68.67 | 0.32 | 32.38~90.71 | 5 | 0 |
| SS | 69.44 | 0.29 | 35.63~84.10 | 5 | 0 |  | 9.59 | 1.41 | -13.12~19.17 | 4 | 1 |

Note: PH: Plant height; EP: Effective number of panicles per plant; GL: Grain length; GW: Grain width; KGW: 1000-grain weight; FGP: Filled grains per plant; TGP: Total grains per plant; GYP: Grain yield per plant; SS: Seed setting. +: the number of hybrids with positive heterosis; - : the number of hybrids with negative heterosis.
