# Supplementary material for: Carbohydrate metabolism and fertility related genes high expression levels promote heterosis in autotetraploid rice harboring double neutral genes
Source: Rice (N Y). 2019 May 10;12:34. doi: 10.1186/s12284-019-0294-x (PMC6510787; doi:10.1186/s12284-019-0294-x)
Supplement: Supplementary file 2 — Table S2. Embryo sac fertility of hybrid and parents. (DOCX 15 kb) [file 12284_2019_294_MOESM2_ESM.docx]

**Table S2.** Embryo sac fertility of hybrid and parents

|  | Total of cell | Number of normal cell | Number of abnormal cell | Normal of embryo sac fertility (%) | Abnormal of embryo sac fertility (%) |
| --- | --- | --- | --- | --- | --- |
| T449 | 208 | 189 | 19 | 90.87 | 9.13 |
| F1 | 157 | 141 | 16 | 89.81 | 10.19 |
| H1 | 243 | 232 | 11 | 95.47 | 4.53 |
