# Supplementary material for: Carbohydrate metabolism and fertility related genes high expression levels promote heterosis in autotetraploid rice harboring double neutral genes
Source: Rice (N Y). 2019 May 10;12:34. doi: 10.1186/s12284-019-0294-x (PMC6510787; doi:10.1186/s12284-019-0294-x)
Supplement: Supplementary file 3 — Figure S1. Chromosome behavior of hybrid. (PPTX 504 kb) [file 12284_2019_294_MOESM3_ESM.pptx]

## Slide 1
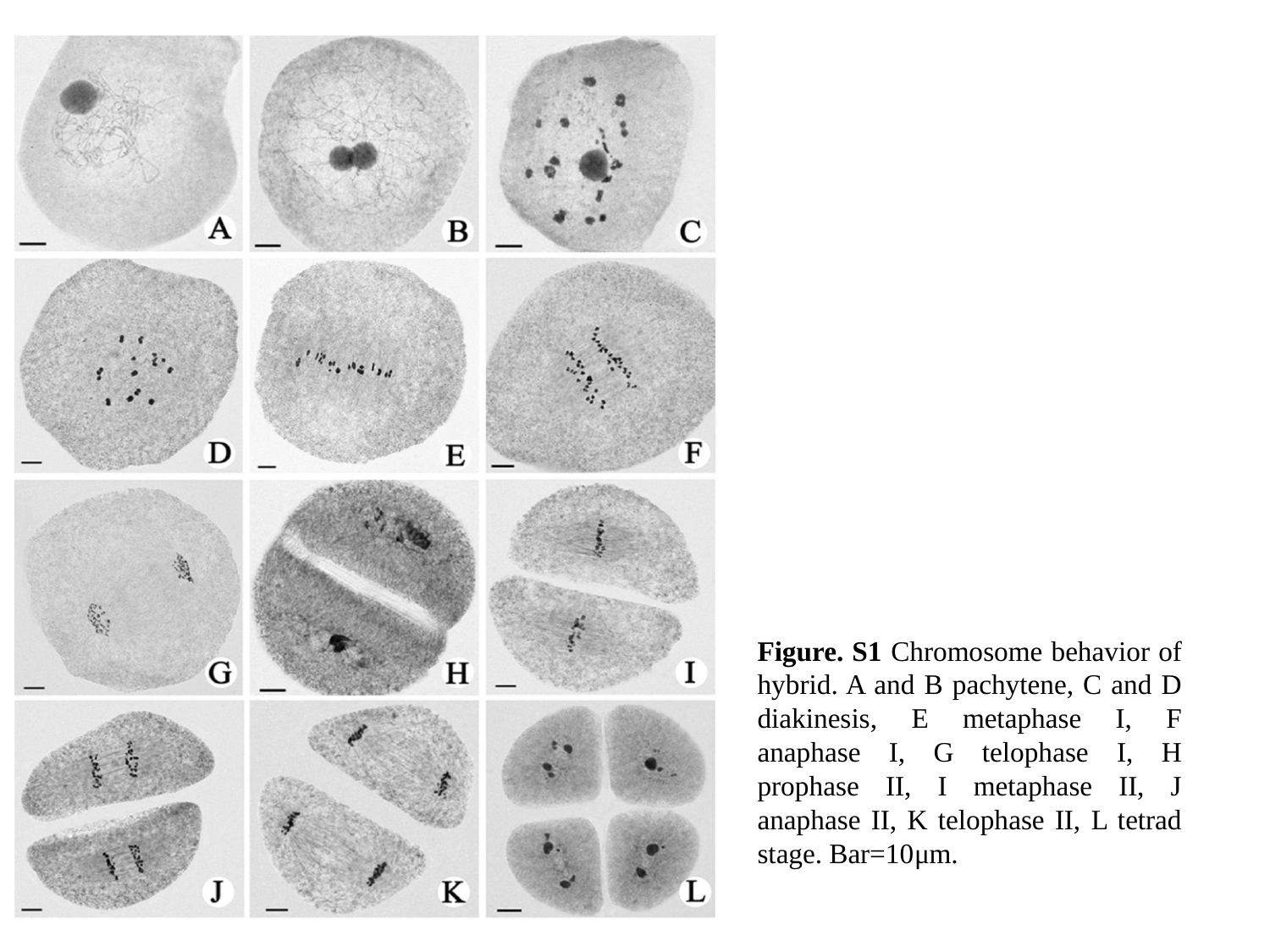

Figure. S1 Chromosome behavior of hybrid. A and B pachytene, C and D diakinesis, E metaphase I, F anaphase I, G telophase I, H prophase II, I metaphase II, J anaphase II, K telophase II, L tetrad stage. Bar=10μm.
