# Supplementary figures and images for: Carbohydrate metabolism and fertility related genes high expression levels promote heterosis in autotetraploid rice harboring double neutral genes
Source: Rice (N Y). 2019 May 10;12:34. doi: 10.1186/s12284-019-0294-x (PMC6510787; doi:10.1186/s12284-019-0294-x)

## Slide 1
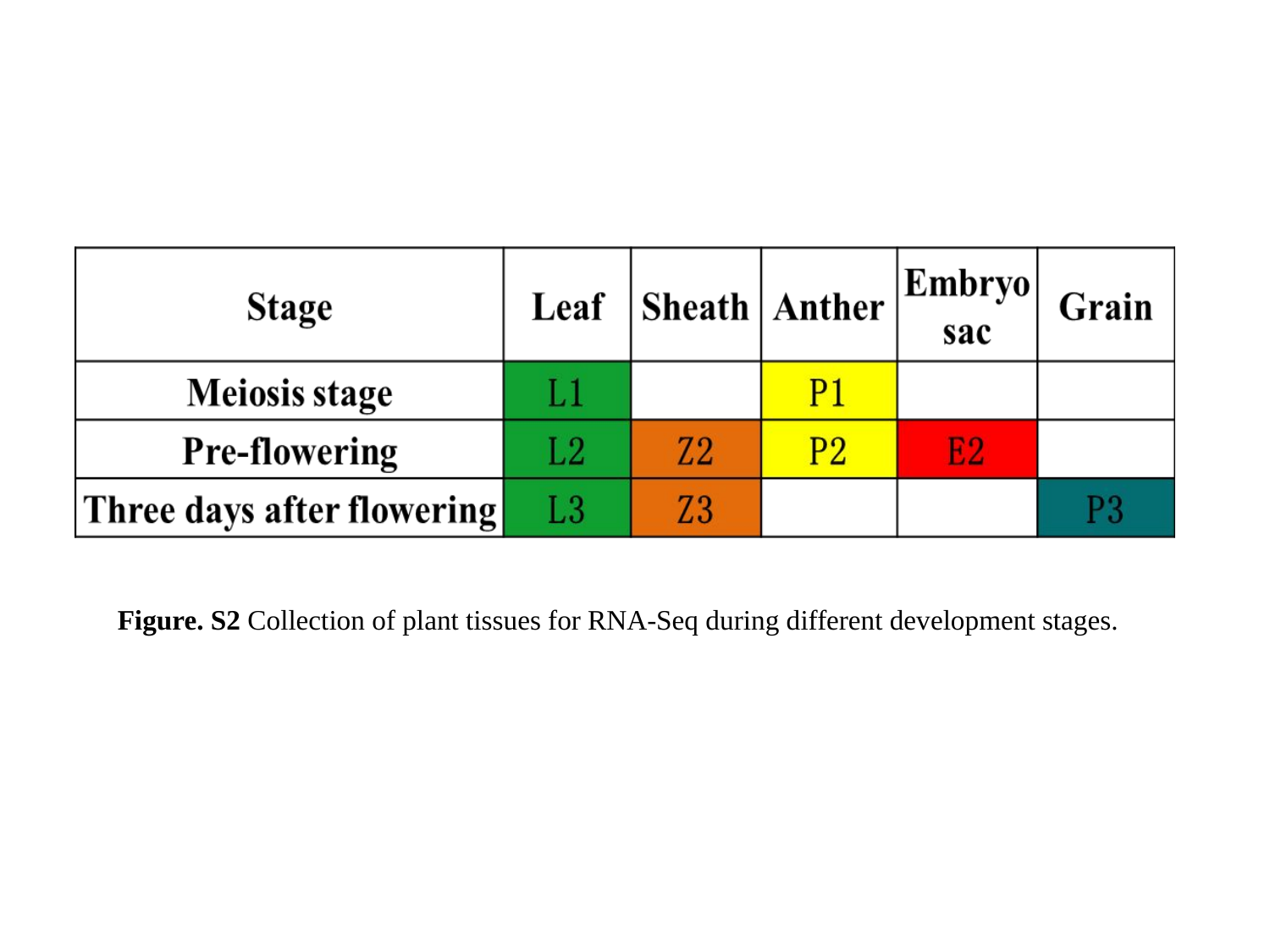

Figure. S2 Collection of plant tissues for RNA-Seq during different development stages.

Supplement: Supplementary file 4 — Figure S2. Collection of plant tissues for RNA-Seq during different development stages. (PPTX 79 kb) [file 12284_2019_294_MOESM4_ESM.pptx]
