# Supplementary material for: Carbohydrate metabolism and fertility related genes high expression levels promote heterosis in autotetraploid rice harboring double neutral genes
Source: Rice (N Y). 2019 May 10;12:34. doi: 10.1186/s12284-019-0294-x (PMC6510787; doi:10.1186/s12284-019-0294-x)
Supplement: Supplementary file 9 — Figure S4. Hierarchical clustering analysis of all gene models based on expression data. (PPTX 557 kb) [file 12284_2019_294_MOESM9_ESM.pptx]

## Slide 1
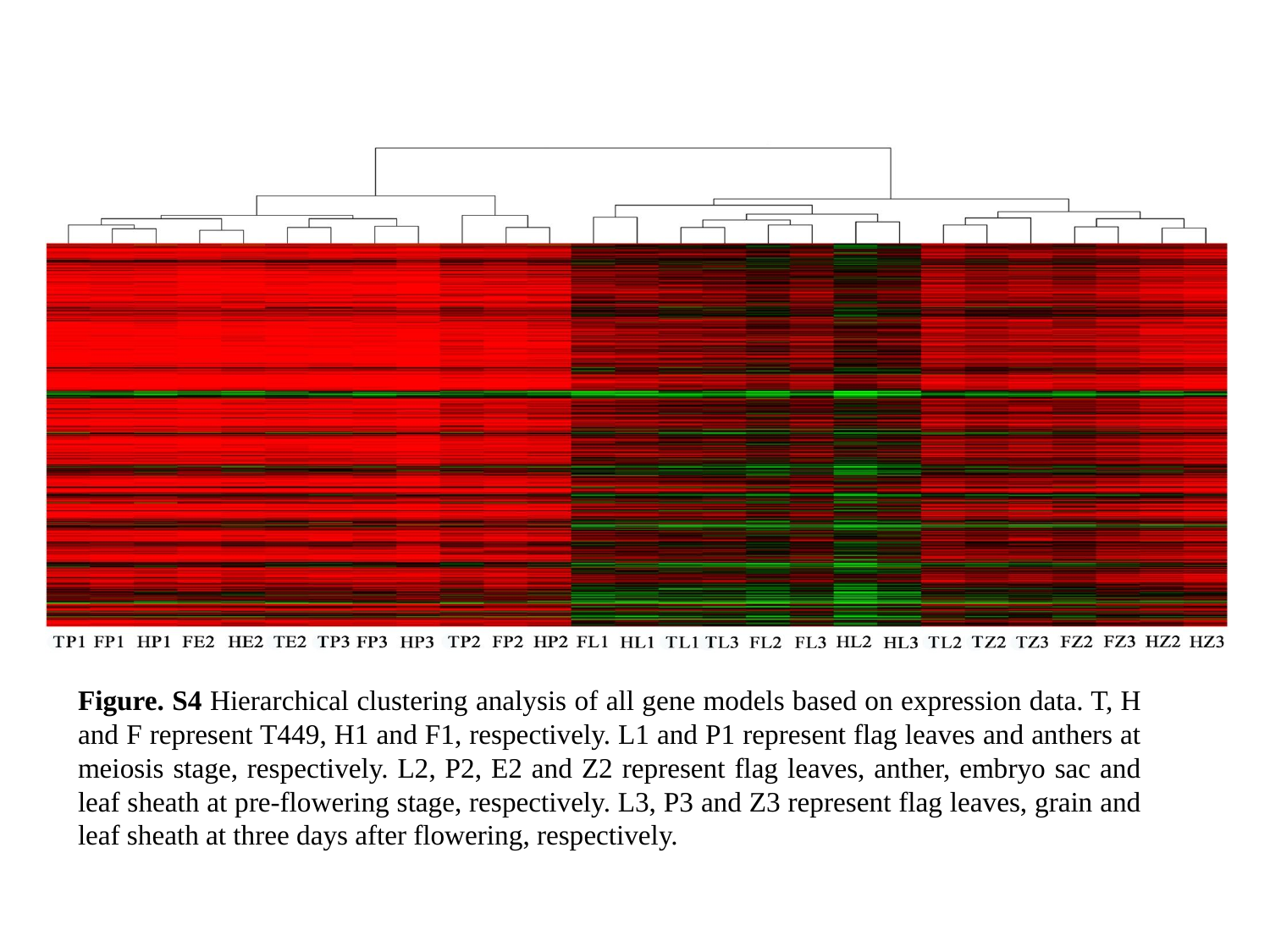

Figure. S4 Hierarchical clustering analysis of all gene models based on expression data. T, H and F represent T449, H1 and F1, respectively. L1 and P1 represent flag leaves and anthers at meiosis stage, respectively. L2, P2, E2 and Z2 represent flag leaves, anther, embryo sac and leaf sheath at pre-flowering stage, respectively. L3, P3 and Z3 represent flag leaves, grain and leaf sheath at three days after flowering, respectively.
