# Supplementary material for: Carbohydrate metabolism and fertility related genes high expression levels promote heterosis in autotetraploid rice harboring double neutral genes
Source: Rice (N Y). 2019 May 10;12:34. doi: 10.1186/s12284-019-0294-x (PMC6510787; doi:10.1186/s12284-019-0294-x)
Supplement: Supplementary file 10 — Figure S5. Comparison of the log2 (FC) of 12 selected transcripts using RNA-Seq and qRT-PCR. (PPTX 90 kb) [file 12284_2019_294_MOESM10_ESM.pptx]

## Slide 1
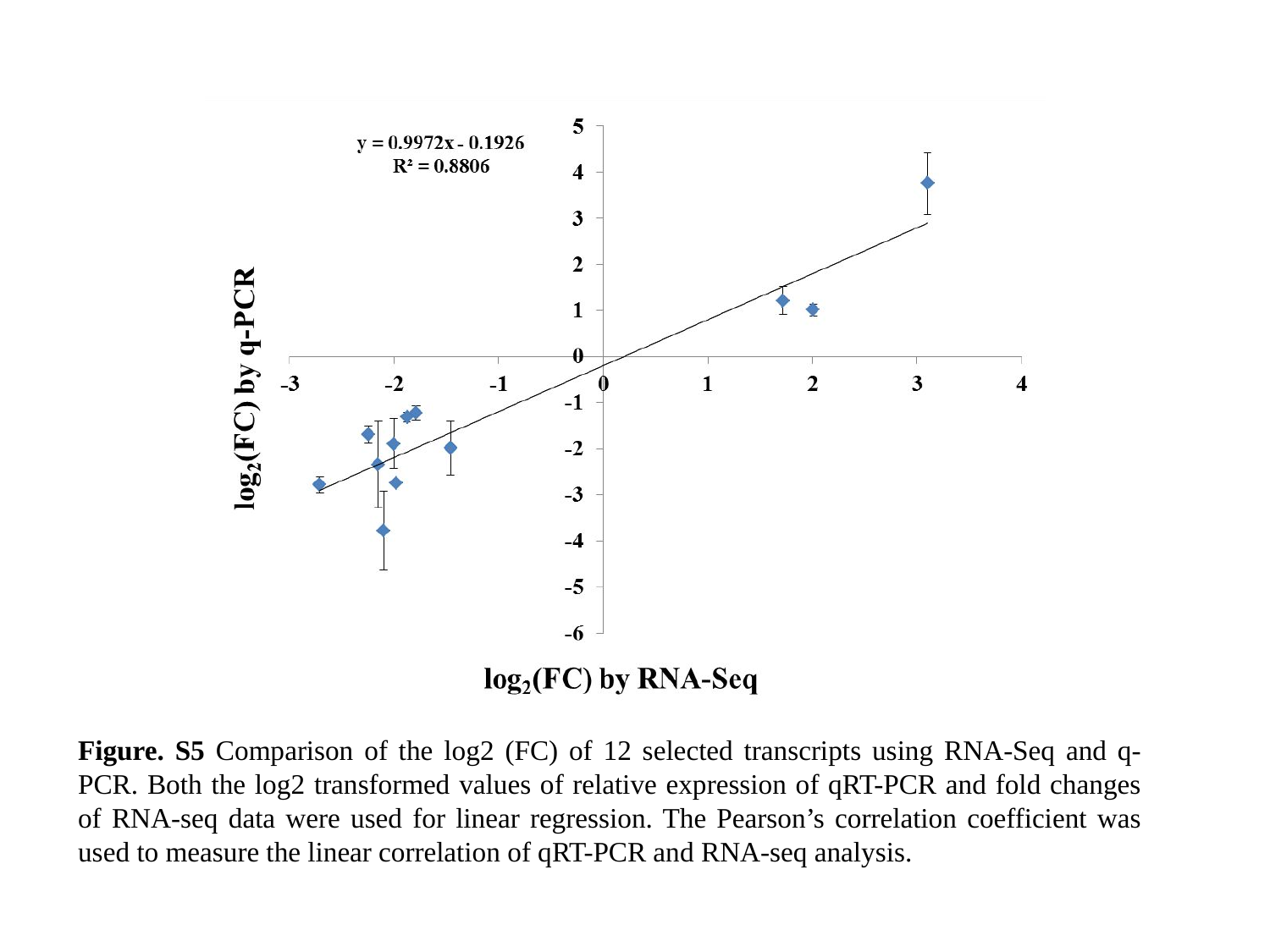

Figure. S5 Comparison of the log2 (FC) of 12 selected transcripts using RNA-Seq and q-PCR. Both the log2 transformed values of relative expression of qRT-PCR and fold changes of RNA-seq data were used for linear regression. The Pearson’s correlation coefficient was used to measure the linear correlation of qRT-PCR and RNA-seq analysis.
