# Supplementary material for: Carbohydrate metabolism and fertility related genes high expression levels promote heterosis in autotetraploid rice harboring double neutral genes
Source: Rice (N Y). 2019 May 10;12:34. doi: 10.1186/s12284-019-0294-x (PMC6510787; doi:10.1186/s12284-019-0294-x)
Supplement: Supplementary file 14 — Figure S7. Distribution of DEGFPU mapped in yield and yield-related QTLs. (PPTX 1011 kb) [file 12284_2019_294_MOESM14_ESM.pptx]

## Slide 1
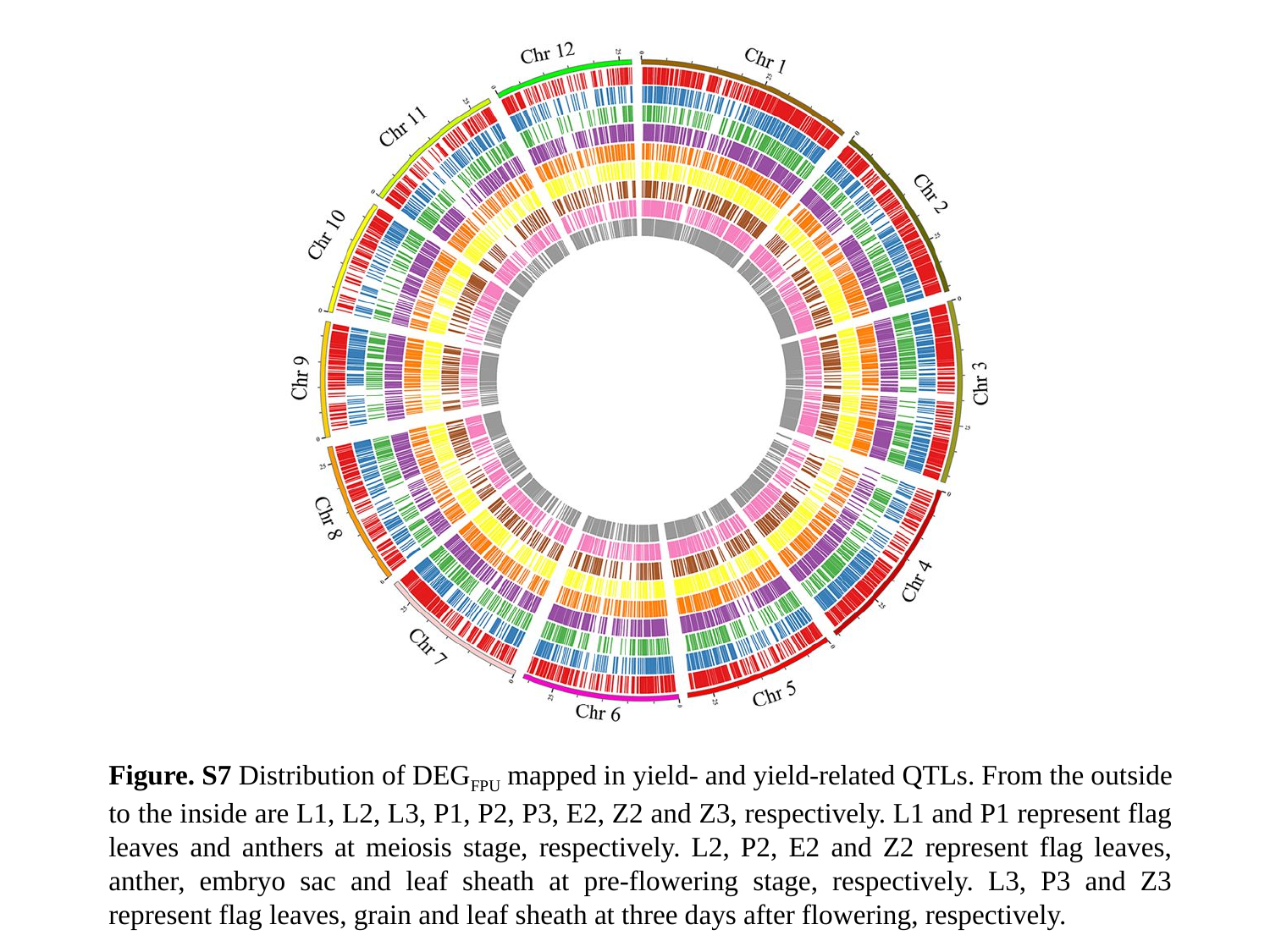

Figure. S7 Distribution of DEGFPU mapped in yield- and yield-related QTLs. From the outside to the inside are L1, L2, L3, P1, P2, P3, E2, Z2 and Z3, respectively. L1 and P1 represent flag leaves and anthers at meiosis stage, respectively. L2, P2, E2 and Z2 represent flag leaves, anther, embryo sac and leaf sheath at pre-flowering stage, respectively. L3, P3 and Z3 represent flag leaves, grain and leaf sheath at three days after flowering, respectively.
