# Supplementary material for: Carbohydrate metabolism and fertility related genes high expression levels promote heterosis in autotetraploid rice harboring double neutral genes
Source: Rice (N Y). 2019 May 10;12:34. doi: 10.1186/s12284-019-0294-x (PMC6510787; doi:10.1186/s12284-019-0294-x)
Supplement: Supplementary file 15 — Figure S8. Distribution of DEGFPU on yield-related QTLs. (PPTX 241 kb) [file 12284_2019_294_MOESM15_ESM.pptx]

## Slide 1
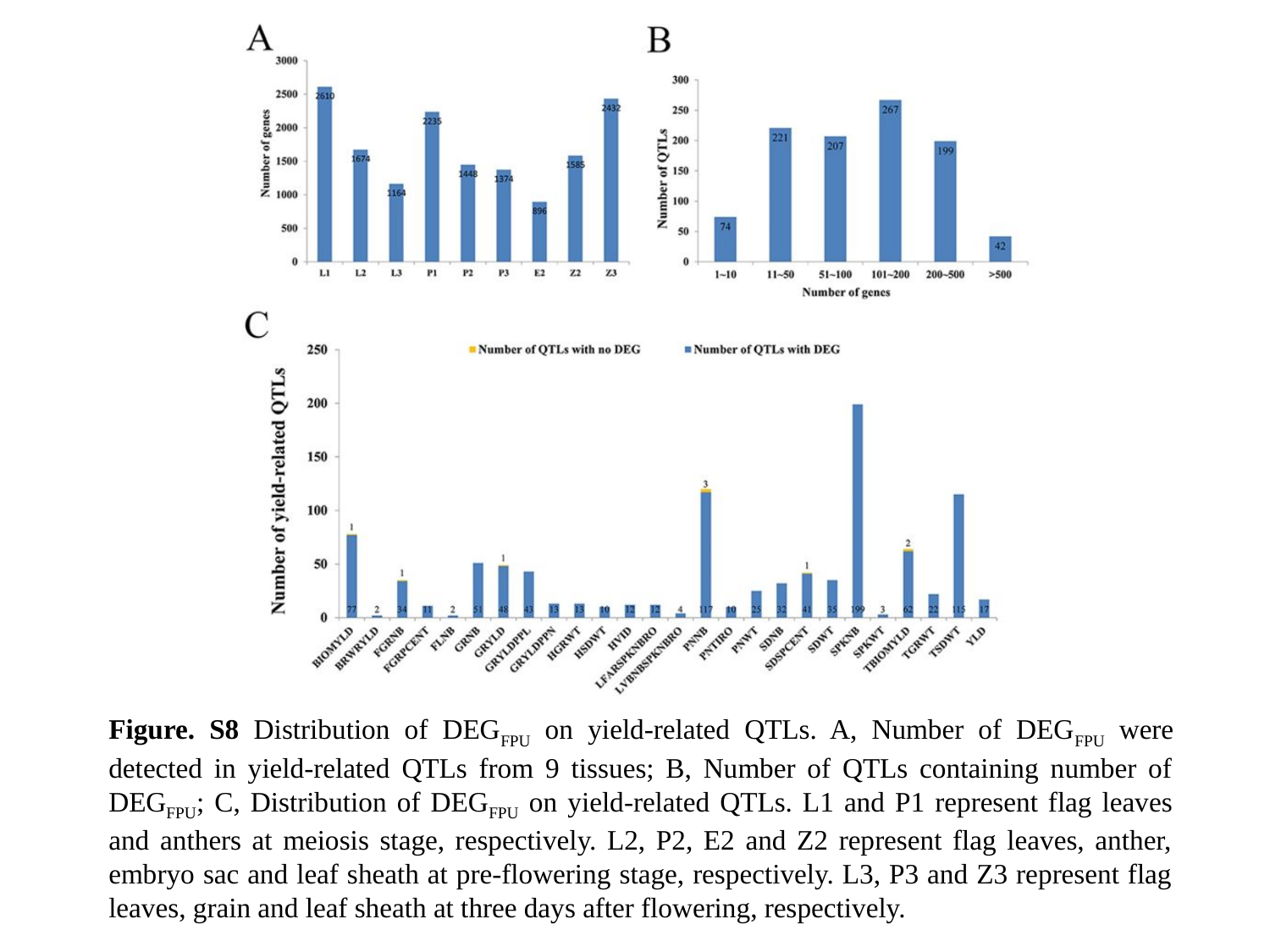

Figure. S8 Distribution of DEGFPU on yield-related QTLs. A, Number of DEGFPU were detected in yield-related QTLs from 9 tissues; B, Number of QTLs containing number of DEGFPU; C, Distribution of DEGFPU on yield-related QTLs. L1 and P1 represent flag leaves and anthers at meiosis stage, respectively. L2, P2, E2 and Z2 represent flag leaves, anther, embryo sac and leaf sheath at pre-flowering stage, respectively. L3, P3 and Z3 represent flag leaves, grain and leaf sheath at three days after flowering, respectively.
