# Supplementary material for: Carbohydrate metabolism and fertility related genes high expression levels promote heterosis in autotetraploid rice harboring double neutral genes
Source: Rice (N Y). 2019 May 10;12:34. doi: 10.1186/s12284-019-0294-x (PMC6510787; doi:10.1186/s12284-019-0294-x)
Supplement: Supplementary file 17 — Table S9. GO analysis of common up-regulated genes between F1 vs T449 and H1 vs T449 in anther at meiosis stage. (DOCX 17 kb) [file 12284_2019_294_MOESM17_ESM.docx]

**Table S9.** GO analysis of common up-regulated genes between F_1_ vs T449 and H1 vs T449 in anther at meiosis stage

| GO term | Ontology | Description | Number in input list | FDR |
| --- | --- | --- | --- | --- |
| GO:0008219 | P | cell death | 18 | 6.20E-06 |
| GO:0016265 | P | death | 18 | 6.20E-06 |
| GO:0012501 | P | programmed cell death | 18 | 6.20E-06 |
| GO:0006915 | P | apoptosis | 18 | 6.20E-06 |
| GO:0006952 | P | defense response | 15 | 5.80E-05 |
| GO:0006950 | P | response to stress | 16 | 0.023 |
| GO:0017076 | F | purine nucleotide binding | 47 | 0.00059 |
| GO:0030554 | F | adenyl nucleotide binding | 45 | 0.00059 |
| GO:0001883 | F | purine nucleoside binding | 45 | 0.00059 |
| GO:0001882 | F | nucleoside binding | 45 | 0.00059 |
| GO:0005524 | F | ATP binding | 42 | 0.00093 |
| GO:0032559 | F | adenyl ribonucleotide binding | 42 | 0.00093 |
| GO:0032555 | F | purine ribonucleotide binding | 44 | 0.00093 |
| GO:0032553 | F | ribonucleotide binding | 44 | 0.00093 |
| GO:0000166 | F | nucleotide binding | 49 | 0.00098 |
