# Supplementary material for: Carbohydrate metabolism and fertility related genes high expression levels promote heterosis in autotetraploid rice harboring double neutral genes
Source: Rice (N Y). 2019 May 10;12:34. doi: 10.1186/s12284-019-0294-x (PMC6510787; doi:10.1186/s12284-019-0294-x)
Supplement: Supplementary file 22 — Table S14. Summary of general sequencing data of maternal line (T449) and paternal line (H1) mapped onto Nipponbare reference genome. (DOCX 16 kb) [file 12284_2019_294_MOESM22_ESM.docx]

**Table S14.** Summary of general sequencing data of maternal line (T449) and paternal line (H1) mapped onto Nipponbare reference genome

| Items | T449 | H1 |
| --- | --- | --- |
| Clean reads | 100395344 | 1.51E+08 |
| Mapped(%) | 98.16 | 96.11 |
| Properly mapped (%) | 94.49 | 90.46 |
| Percent of bases ≥Q30 (%) | 86.81 | 92.28 |
| GC content (%) | 45.1 | 44.55 |
| Coverage ratio 10x (%) | 93.75 | 92.15 |
| Average coverage depth | 35 | 49 |
