# Supplementary material for: Carbohydrate metabolism and fertility related genes high expression levels promote heterosis in autotetraploid rice harboring double neutral genes
Source: Rice (N Y). 2019 May 10;12:34. doi: 10.1186/s12284-019-0294-x (PMC6510787; doi:10.1186/s12284-019-0294-x)
Supplement: Supplementary file 23 — Table S15. Effect type annotation and distribution of SNPs and InDels in different genomic regions. (DOCX 18 kb) [file 12284_2019_294_MOESM23_ESM.docx]

**Table S15.** Effect type annotation and distribution of SNPs and InDels in different genomic regions

| Class | SNP | |  | Class | InDels | |
| --- | --- | --- | --- | --- | --- | --- |
|  | No | rate（%） |  |  | No | rate（%） |
| UPSTREAM | 314687 | 34.4714 |  | UPSTREAM | 73282 | 37.3934 |
| DOWNSTREAM | 239620 | 26.2484 |  | DOWNSTREAM | 55273 | 28.204 |
| INTERGENIC | 49680 | 5.442 |  | INTERGENIC | 11580 | 5.9089 |
| INTRAGENIC | 40 | 0.0044 |  | INTRAGENIC | 56 | 0.0286 |
| INTRON | 149939 | 16.4246 |  | INTRON | 35809 | 18.2721 |
| SPLICE_SITE_ACCEPTOR | 500 | 0.0548 |  | SPLICE_SITE_ACCEPTOR | 103 | 0.0526 |
| SPLICE_SITE_DONOR | 426 | 0.0467 |  | SPLICE_SITE_DONOR | 106 | 0.0541 |
| SPLICE_SITE_REGION | 3295 | 0.3609 |  | SPLICE_SITE_REGION | 632 | 0.3225 |
| START_GAINED | 1390 | 0.1523 |  | FRAME_SHIFT | 4953 | 2.5274 |
| START_LOST | 267 | 0.0292 |  | START_LOST | 50 | 0.0255 |
| STOP_GAINED | 2944 | 0.3225 |  | STOP_GAINED | 105 | 0.0536 |
| STOP_LOST | 481 | 0.0527 |  | STOP_LOST | 49 | 0.025 |
| UTR_3_PRIME | 17019 | 1.8643 |  | UTR_3_PRIME | 5719 | 2.9182 |
| UTR_5_PRIME | 7292 | 0.7988 |  | UTR_5_PRIME | 3976 | 2.0288 |
| NON_SYNONYMOUS_CODING | 75318 | 8.2505 |  | CODON_CHANGE_PLUS_CODON_DELETION | 771 | 0.3934 |
| NON_SYNONYMOUS_START | 20 | 0.0022 |  | CODON_CHANGE_PLUS_CODON_INSERTION | 433 | 0.2209 |
| SYNONYMOUS_CODING | 49896 | 5.4657 |  | CODON_DELETION | 1394 | 0.7113 |
| SYNONYMOUS_STOP | 78 | 0.0085 |  | CODON_INSERTION | 1684 | 0.8593 |
|  |  |  |  | EXON_DELETED | 1 | 0.0005 |
| Total | 912892 | 1 |  | Total | 195976 | 1 |
