# Supplementary material for: Carbohydrate metabolism and fertility related genes high expression levels promote heterosis in autotetraploid rice harboring double neutral genes
Source: Rice (N Y). 2019 May 10;12:34. doi: 10.1186/s12284-019-0294-x (PMC6510787; doi:10.1186/s12284-019-0294-x)
Supplement: Supplementary file 27 — Table S19. List of primers used for qRT-PCR. (DOCX 16 kb) [file 12284_2019_294_MOESM27_ESM.docx]

**Table S19.** List of primers used for qRT-PCR

| Name | Forward primer | Reverse primer |
| --- | --- | --- |
| LOC_Os10g38110 | CAACACCAAGGCACACGACA | ACAGGAACCAAGCAAGCGA |
| LOC_Os09g36220 | ACGCATGCAAGAATATCCCC | CAGCTTTCGTCACTCCGGTC |
| LOC_Os02g41650 | GTGATGCAGGTGGCCAAGAAAT | GAGCTTCTGCATCAACGGGTA |
| LOC_Os03g28940 | GGATGCGAGGGAGCAAGAGA | GCGGAAGATGGAAGAAGGACA |
| LOC_Os04g41500 | ATCTGGTCTGGCTGGGATTT | CGGGGCTAACAGTTGTCGT |
| LOC_Os03g39830 | GGTGGAGATGGGGAGGTGAA | CCAAAGATGATGATGTAGGGCA |
| LOC_Os02g15750 | ACCACATTGGTCATCCTTGACA | CATCCCCGATTAGATCGCTC |
| LOC_Os05g05140 | ACTGGCTTGGCTGTTCTTCTC | TGTGTCTCGCAATCCGTTTAC |
| LOC_Os07g49400 | CCAACTTCCCATCCTCTCCTA | TGTCCTTGTCACTCAAACCCA |
| LOC_Os04g45490 | CCATCACCACTTGACCTACCAC | GCCTTCCGATTCTTTCCTTCTT |
| LOC_Os04g37619 | GGAAGACCGTTAAGCTGTAGGC | CAGAACTTGTGGGGAGGAGG |
| LOC_Os07g47510 | TCAACAACCTGCCGCTGTT | TTCGCCGACTCGCTCTTC |
| ubiquitin | CAAGATGATCTGCCGCAAATGC | TTTAACCAGTCCATGAACCCG |
